# In Silico and In Vivo Evaluation of Novel 2-Aminobenzothiazole Derivative Compounds as Antidiabetic Agents

**DOI:** 10.3390/ijms26030909

**Published:** 2025-01-22

**Authors:** Juan Andres Alvarado Salazar, Miguel Valdes, Alejandro Cruz, Brenda Moreno de Jesús, David Patiño González, Ivonne María Olivares Corichi, Feliciano Tamay Cach, Jessica Elena Mendieta Wejebe

**Affiliations:** 1 Carrera de Química Farmacéutica Biológica, Área Farmacéutica, Facultad de Estudios Superiores (FES)-Zaragoza, Universidad Nacional Autónoma de México, Batalla 5 de mayo s/n, Ejercito de Oriente, Iztapalapa, Mexico City 09230, Mexico; andres.alvarado.salazar@comunidad.unam.mx; 2 Laboratorio de Biofísica y Biocatálisis, Sección de Estudios de Posgrado e Investigación, Escuela Superior de Medicina, Instituto Politécnico Nacional, Av. Salvador Díaz Mirón esq. Plan de San Luis s/n, Casco de Santo Tomás, Miguel Hidalgo, Mexico City 11340, Mexico; bmoj0412@gmail.com (B.M.d.J.); davidyoto16@gmail.com (D.P.G.); 3 Unidad de Investigación Médica en Farmacología, UMAE Hospital de Especialidades 2° Piso CORSE Centro Médico Nacional Siglo XXI, Instituto Mexicano del Seguro Social, Av. Cuauhtémoc 330, Col. Doctores, Mexico City 06720, Mexico; 4 Laboratorio de Química Supramolecular y Nanociencias, Departamento de Ciencias Básicas, Unidad Profesional Interdisciplinaria de Biotecnología, Instituto Politécnico Nacional, Av. Acueducto s/n, Colonia Barrio La Laguna Ticomán, Mexico City 07340, Mexico; alecruz@ipn.mx; 5 Laboratorio de Bioquímica y Estrés Oxidante, Sección de Estudios de Posgrado e Investigación, Escuela Superior de Medicina, Instituto Politécnico Nacional, Av. Salvador Díaz Mirón esq. Plan de San Luis s/n, Casco de Santo Tomás, Miguel Hidalgo, Mexico City 11340, Mexico; iolivares@ipn.mx; 6 Laboratorio de Investigación de Bioquímica Aplicada, Sección de Estudios de Posgrado e Investigación, Escuela Superior de Medicina, Instituto Politécnico Nacional, Av. Salvador Díaz Mirón esq. Plan de San Luis s/n, Casco de Santo Tomás, Miguel Hidalgo, Mexico City 11340, Mexico; ftamay@ipn.mx

**Keywords:** 2-aminobenzothiazole, isothioureas, guanidines, type 2 diabetes, antidiabetic activity

## Abstract

Currently, there are several drugs used for the treatment of type 2 diabetes (T2D); however, all of them have adverse effects. Benzothiazoles have a broad spectrum of biological activities such as antidiabetic. This study aimed to evaluate in silico and in vivo two series of 2-aminobenzothiazole derivatives linked to isothioureas (**3a**–**w**) or guanidines (**4a**–**z**) for the treatment of T2D. The ADMET properties were determined in silico, from which it was possible to select nine compounds (two isothioureas and seven guanidines), and, with molecular docking, it was shown that compounds methyl (E)-N′-(benzo[d]thiazol-2-yl)-N-methylcarbamimidothioate (**3b**) and 2-(benzo[d]thiazol-2-yl)-1,3-di-tert-butylguanidine (**4y**) showed a high affinity for PPARγ (ΔG = −7.8 and −8.4 kcal/mol, respectively). In vivo, the LD50 value was estimated in rats based on OECD Guideline 425, being >1750 mg/kg for both compounds. The pharmacological effect of **3b** and **4y** was evaluated in the T2D rat model, showing that after oral administration in an equimolar ratio to pioglitazone (15 mg/kg) for 4 weeks, both compounds were able to reduce blood glucose levels (<200 mg/dL) and improve the lipid profile. Therefore, **3b** and **4y** could be used in the future as antidiabetic agents.

## 1. Introduction

Diabetes is a chronic and progressive endocrine disease characterized by uncontrolled hyperglycemia, which is associated with poor pancreatic function in the synthesis and secretion of insulin and an inadequate response of peripheral tissues to the action of this hormone, which leads to a series of metabolic alterations that cause cellular dysfunction [1]. Over time, these pathological processes lead to the development of complications that can cause serious health problems [2,3,4,5,6,7,8,9,10]. In 2021, the global prevalence of people aged 20 to 79 years diagnosed with diabetes was estimated at 10.5% (536.6 million) and is expected to increase to 12.2% (783.2 million) by 2045 [11]. It is known that in low- and middle-income countries, there is a higher prevalence and mortality of people with diabetes [11,12,13].

Because most patients with diabetes have type 2 diabetes (T2D), this disease has become a challenge for the scientific community [5,14]. Pharmacological therapies available for the treatment of T2D include oral hypoglycemic agents, the most used being biguanides, sulfonylureas, thiazolidinediones, and alpha-glucosidase inhibitors. Other therapies include glucagon-like peptide-1 agonists, dipeptidyl peptidase-4 inhibitors, sodium–glucose cotransporter-2 inhibitors, and insulin [4,15,16,17,18]. These drugs can be administered as monotherapy, but considering that T2D is a progressive disease, its management in more advanced stages requires the combination of several oral hypoglycemic agents to maintain the efficacy of the treatment [19]. However, this leads to an increasing profile of side effects such as digestive disturbances (nausea and diarrhea), weight gain, hypoglycemia, anemia, neuropathy, hepatotoxicity, cardiovascular risk, and bladder cancer, in which the incidence or severity of damage to health will depend on each of them, which ultimately becomes a limiting factor of the treatment [9,20]. Therefore, there is a need to search for alternative therapeutic agents for the treatment of T2D, which are based on the use of structural prototypes for the development of new chemical entities with potential antidiabetic activity [7,21]. In this regard, it has been reported that various compounds incorporating heterocyclic and fused heterocyclic rings play an important role in the design and synthesis of antidiabetic agents; mainly those containing nitrogen, sulfur, and oxygen atoms have attracted medical interest due to their countless and promising pharmaceutical applications [22,23].

Benzothiazoles are compounds belonging to the class of bicyclic heteroaromatics, which have shown a broad spectrum of biological activities, such as antimicrobial, anticancer, and antidiabetic, among others [24,25,26,27,28]. There are several commercially available drugs that contain the benzothiazole nucleus, such as etoxzolamide, which is used in heart diseases, glaucoma, and more recently as an antimicrobial agent [29,30,31,32]. Among the derivatives of the 2-aminobenzothiazole nucleus is riluzole, which is useful in the treatment of amyotrophic lateral sclerosis [33,34], while frentizole has antiviral and immunosuppressive properties [23,35], although its pharmacological effect has also been identified in neurodegenerative disorders [36,37,38]. On the other hand, zopolrestat was developed by Pfizer for the treatment of diabetic complications [22,39]. In this regard, several studies have shown that the antidiabetic activity of different series of compounds derived from the benzothiazole nucleus is associated with their agonist effect on the peroxisome proliferator-activated receptor (PPAR) [24,40,41,42,43,44,45,46,47].

The PPAR is a member of the nuclear receptor superfamily that acts as a ligand-dependent transcription factor modulating the up- or down-regulation of genes involved in the activation of intra- and extracellular signaling pathways. Currently, three isoforms of this receptor are known: PPAR alpha (PPARα), PPAR beta/delta (PPARβ/δ), and PPAR gamma (PPARγ), which is considered a key molecular target in the control of T2D due to its important role in promoting glucose uptake in peripheral tissues, adipocyte remodeling, and insulin sensitivity, among other metabolic and non-metabolic functions [48]. Recent research has focused on finding ligands with dual- or pan-PPAR agonist effects to provide patients with access to drugs with more beneficial effects (e.g., normalization of insulin resistance, reduction of plasma lipids, etc.) and fewer adverse effects (e.g., slowing of heart failure progression) compared to isoform-selective ligands (PPARα, PPARβ/δ, or PPARγ) [48,49,50,51]. However, very few dual- or pan-PPAR ligands are currently in the different development phases in pharmaceutical companies, with benzafibrate being the first and only pan-PPAR agonist used therapeutically [48]. For this reason, the design and evaluation of new selective PPAR ligands remains a useful strategy for the treatment of T2D and its micro- and macrovascular complications, as well as other diseases. An example of this is pioglitazone (PGZ), which is a selective PPARγ agonist that, in addition to being used as an insulin sensitizer for the treatment of metabolic syndrome and T2D, has also been shown to be effective in non-alcoholic fatty liver disease, chronic kidney disease, inflammatory and autoimmune diseases, as well as central nervous system disorders and depression. These effects are exerted by PGZ by activating different molecular targets and signaling pathways [52]. In this regard, it is worth mentioning that most of the benzothiazole nucleus derivatives that have been evaluated so far have shown a selective agonist effect on PPARγ [24,40,42,43,46].

**Figure 1:**
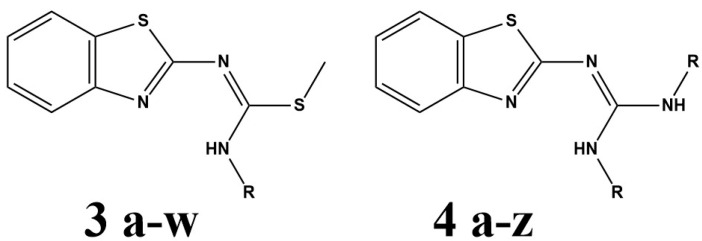
General chemical structure of 2-aminobenzothiazole derivatives linked to different isothioureas (**3a**–**w**) or guanidines (**4a**–**z**).

Therefore, this study aimed to evaluate the antidiabetic activity of hybrid compounds derived from the 2-aminobenzothiazole nucleus linked to different isothioureas (**3a**–**w**) or guanidines (**4a**–**z**) (Figure 1) in a rat model with T2D, which were previously designed and synthesized by our research group [53,54,55,56,57,58].

## 2. Results and Discussion

### 2.1. In Silico Studies

#### 2.1.1. ADMET Properties

Forty-nine molecules derived from the 2-aminobenzothiazole nucleus linked to isothioureas (**3a**–**w**) or guanidines (**4a**–**z**) were designed (Figure 1). Subsequently, their physicochemical properties and toxicity parameters were determined, which allowed the first screening to be carried out, obtaining a total of 28 compounds. The second screening was carried out by calculating the parameters of aqueous solubility, pharmacokinetics, and medicinal chemistry. Finally, the third screening was carried out considering the value of the free binding energy (ΔG) obtained from the molecular docking, with which nine compounds with very high potential for the treatment of T2D were chosen. The results obtained from this first in silico study are presented below, considering the data from the nine compounds chosen as the most promising up to that point (Table 1, Table 2, Table 3 and Table 4). The way in which the score was assigned in each screening from the 49 compounds is described in the Supplementary Materials (Tables S1–S4).

The physicochemical properties and toxicity parameters of the evaluated compounds, which were evaluated by Molinspiration Cheminformatics (https://www.molinspiration.com/, accessed on 21 September 2022) [59] and DataWarrior (https://openmolecules.org/datawarrior/, accessed on 25 September 2022) [60,61], are presented in Table 2. This set of data is known as Lipinski and Veber rules [62,63,64], which must be fulfilled by new chemical entities to be considered as potential oral drugs. The molecular weight (MW) should be less than 500 g/mol, the water–octanol partition coefficient (cLogP) should be less than 5, which indicates that it has adequate liposolubility through the cell membrane, it should not have more than 5 donors (nOHNH) or more than 10 acceptors (nON) of hydrogen bonds, the number of rotatable angles (nrotb) should be less than 10, and the topological polar surface area (TPSA) should be less than 140 Å^2^, since it represents adequate permeability in cells of the digestive tract, while if the value is less than 90 Å^2^, then a good penetration of the blood–brain barrier (BBB) can be assumed [62,63,64,65,66,67].

As can be observed in Table 2, most of the compounds satisfactorily comply with Lipinski’s rule of five [62,63] and the considerations of Veber et al. [64], except for **4y**, which has a cLogP value greater than 5, which is not very advisable considering that this parameter is associated with hydrophobicity that affects drug absorption, bioavailability, hydrophobic ligand–protein interactions, metabolism of the compounds, as well as their toxicity. However, **4y** was not discarded from this first virtual screening even though it would be advisable to make some structural modifications to this compound in the future. In addition, according to the predictors used, it is suggested that none of the evaluated compounds is associated with mutagenicity, tumorigenicity, and/or irritant and reproductive effects. Based on these results, in the last column of Table 2 it is possible to observe the value of the overall drug similarity score (ODLS), which qualitatively evaluates the possibility of a compound becoming an orally administered drug with respect to its bioavailability [64].

Subsequently, the compounds with an ODLS of 1.0 were determined for their toxicological properties using ProTox 3.0 (https://tox.charite.de/protox3/, accessed on 5 October 2022) [68,69], which are presented in Table 3. The results show that all compounds belong to Class IV (300 < LD50 ≤ 2000 mg/kg), indicating that they produce low toxicity according to the Globally Harmonized System of Classification and Labeling of Chemicals (https://unece.org/about-ghs, accessed on 5 October 2022) [68,69,70]. Therefore, since the LD50 value is high (>1750 mg/kg), the compounds are expected to be quite safe for use as pharmaceuticals.

**Table 3:** Toxicological properties of the best benzothiazolisothioureas (**3**) and guanidinobenzothiazoles (**4**) obtained from ProTox 3.0 (https://tox.charite.de/protox3/, accessed on 5 October 2022) [68,69].

Compound	LD50 (mg/kg)	Class	Hepatotoxicity	Immunotoxicity	Cytotoxicity
**3a**	1190	IV	X	X	X
**3b**	1000	IV	X	X	X
**4a**	1190	IV	X	X	X
**4b**	1190	IV	X	X	X
**4c**	1190	IV	X	X	X
**4r**	1190	IV	X	X	X
**4s**	1190	IV	X	X	X
**4x**	1190	IV	X	X	X
**4y**	1190	IV	X	X	X
PGZ	1000	IV	X	X	X

Abbreviations and definitions: **PGZ:** pioglitazone. **LD50:** median lethal dose (the estimated dose required to kill 50% of a group of experimental animals). **Hepatotoxicity:** injury to the liver or impairment of the liver function caused by exposure to xenobiotics such as drugs, food additives, alcohol, chlorinated solvents, peroxidized fatty acids, fungal toxins, radioactive isotopes, environmental toxicants, and even some medicinal plants. **Immunotoxicity:** adverse effects on the immune system caused by xenobiotics like drugs and chemicals, leading to dysfunction or structural damage, and potentially resulting in immune diseases or increased susceptibility to infections. **Cytotoxicity:** chemical potency that brings variations in cellular functions and results in cell death. **X:** it does not present the toxicological effect. The definitions of toxicological properties were obtained from https://www.sciencedirect.com/topics/ (accessed on 5 October 2022).

**Table 4:** Aqueous solubility, pharmacokinetics, and medicinal chemistry parameters of the best benzothiazolisothioureas (**3**) and guanidinobenzothiazoles (**4**) obtained from SwissADME (http://www.swissadme.ch/, accessed on 12 October 2022) [71,72].

Compound	Aqueous Solubility			Pharmacokinetics													ODLS
	logS	Solubility (mg/mL)	Class	GI		BBB		P-gp	CYP450 Inhibitor							BD	
									1A2	2C19		2C9		2D6	3A4		
**3a**	−3.00	0.224	III	√		X		X	√	√		X		X	X	0.55	0.84
**3b**	−3.24	0.137	III	√		X		X	√	√		√		X	X	0.55	0.80
**4a**	−1.95	2.15	IV	√		X		X	√	X		X		X	X	0.55	0.92
**4b**	−2.19	1.34	III	√		X		X	√	X		X		X	X	0.55	0.88
**4c**	−2.43	0.822	III	√		X		X	√	X		X		X	X	0.55	0.88
**4r**	−4.51	8.31× 10^−3^	II	√		X		X	√	X		X		X	X	0.55	0.84
**4s**	−4.14	1.95 × 10^−2^	II	√		X		X	√	X		X		X	√	0.55	0.80
**4x**	−3.56	7.64 × 10^−2^	III	√		√		X	√	√		√		X	X	0.55	0.80
**4y**	−3.93	3.56 × 10^−2^	III	√		X		X	√	√		√		X	X	0.55	0.80
PGZ	−4.31	1.76 × 10^−2^	II	√		X		X	√	√		√		√	√	0.55	0.68
**Compound**	**Drug-Likeness**								**Medicinal Chemistry**								
	**Lipinski**	**Ghose**	**Veber**		**Egan**		**Muegge**		**PAINS**		**Brenk**		**Synthesis Accessibility**				
**3a**	√	√	√		√		√		0		2		2.83				
**3b**	√	√	√		√		√		0		2		3.03				
**4a**	√	√	√		√		X		0		2		2.50				
**4b**	√	√	√		√		√		0		2		2.69				
**4c**	√	√	√		√		√		0		2		2.77				
**4r**	√	√	√		√		√		0		0		3.22				
**4s**	√	√	√		√		√		0		0		2.86				
**4x**	√	√	√		√		√		0		2		3.16				
**4y**	√	√	√		√		√		0		2		3.38				
PGZ	√	√	√		√		√		0		1		3.46				

Abbreviations and definitions: **logS:** logarithm of the aqueous solubility (key physicochemical attribute required for the characterization of an active pharmaceutical ingredient during drug discovery and beyond. Furthermore, aqueous solubility is highly important for formulation selection and subsequent development processes. **Class I:** poorly soluble; **Class II:** moderately soluble; **Class III:** soluble; **Class IV:** very soluble; **Class V:** highly soluble). **GI:** gastrointestinal absorption (related to the space that the drug can occupy in the absorption processes prior to entering the disposition space via the first pass through the liver. **√:** high). **BBB:** blood–brain barrier permeability (related to a physical barrier in the central nervous system that regulates the entry of molecules from the bloodstream to the brain, protecting it from harmful substances). **P-gp:** P-glycoprotein substrate (ATP-dependent cellular transporter proteins that work for the elimination of xenobiotic compounds, specifically from intracellular sites to extracellular locations across the cell membrane). **CYP450:** cytochrome P-450 (family of enzymes that play a crucial role in the metabolism of drugs and other foreign substances in the body. These enzymes carry out chemical reactions such as hydroxylation and oxidation, making them important for studying drug interactions due to the inhibition of one or more of their isoforms). **BD:** bioavailability (rate and extent to which the unchanged drug reaches the systemic circulation and consequently at the site of action). **√:** it does present activity; **X:** it does not present activity. Lipinski’s rule of five (Pfizer), Ghose filter, Veber filter, Egan filter (Pharmacia), Muegge filter (Bayer): **√:** it meets all parameters; **X:** it does not meet all parameters. **PAINS:** interference structures in panel assays (structures that may present a false positive in activity). **Brenk:** structural alert (depict fragments of compounds that could be putatively toxic, chemically reactive, and metabolically unstable). **Synthesis accessibility:** 1 easy–10 difficult. The definitions of aqueous solubility, pharmacokinetics, and medicinal chemistry parameters were obtained from https://www.sciencedirect.com/topics (accessed on 12 October 2022).

Table 4 presents the aqueous solubility, pharmacokinetic, and medicinal chemistry parameters of the compounds analyzed in SwissADME (http://www.swissadme.ch/, accessed on 12 October 2022) [71,72]. Aqueous solubility is a characteristic of great pharmaceutical importance for compounds proposed as potential enteral medications, since this property facilitates the handling and formulation of these compounds and influences their intestinal absorption. In this regard, the compound considered highly soluble in aqueous medium (Class IV) is **4a**, while the moderately soluble (Class II) are **4r** and **4s**. The rest of the evaluated compounds present a medium solubility (Class III).

On the other hand, all compounds show a high gastrointestinal (GI) absorption, indicating that they are susceptible to the first-pass effect, while the only compound that can cross the BBB is **4x**. Within the pharmacokinetic parameters, it is of great value to know whether or not a compound is a substrate of P-glycoprotein (P-gp), which is the most important in the function of ATP-dependent transporters (ABC), since it stands out for its multiple functions in the digestive tract, in the central nervous system and also because it is overexpressed in different types of cancer leading to resistance to multiple drugs [73,74]. In the case of the compounds presented in Table 4, it is predicted that none of them is a substrate of P-gp. Similarly, it is essential to understand the interaction of xenobiotics with cytochrome P450 (CYP450), which is the main superfamily of isoenzymes responsible for the elimination of drugs through their biotransformation into water-soluble (polar) compounds. However, the inhibition of one or more CYP450 isoenzymes is related to various drugs that have toxic effects and lead to adverse effects. According to Di, 50 to 90% of drugs interact with five mains human CYP450 isoforms (1A2, 2C19, 2C9, 2D6, 3A4) and the inhibition of any of these leads to adverse effects due to toxicity [75]. Therefore, it is of great importance to highlight that none of the compounds evaluated inhibits all CYP450 isoforms, unlike drugs such as PGZ, which, by inhibiting the five CYP450 isoforms, has been shown to cause ventricular hypertrophy and hepatic and renal congestion in Swiss albino mice [76].

In addition to using pharmacokinetic and toxicological parameters and Lipinski’s rule of five [62,63], major pharmaceutical companies have established various filters to have reliable and high-quality chemical compound libraries. In this sense, the improvements that have been made to Lipinski’s rule of five were published in 1999 as the Ghose rules (Amgen Inc.), which establish that the cLogP value must be between −0.4 and +5.6, the MW must be greater than 160 and less than 500 g/mol, the molar refractivity must have a value between 40 and 130, and the total number of atoms must be between 20 and 70. For its part, Veber’s rules [64] indicate that molecules must have an TPSA value less than 140 Å^2^ and the nrotb must be less than 10. Egan’s rules, established by the pharmaceutical and biotechnology company Pharmacia, establish that the allowed TPSA value must be less than 136.1 Å^2^ and the cLogP less than 5.88 to consider a chemical entity as a potential drug. Muegge’s rules state that the MW must be greater than 200 and less than 600 g/mol, the cLogP value must be between −2 and 5, the TPSA must be less than 150 Å^2^, the number of rings in a structure must be less than 7, the number of carbon atoms must be greater than 5 and the number of heteroatoms greater than 1, it must not have an nrotb greater than 15, and the hydrogen acceptors and donors that can form hydrogen bonds must not exceed 10 and 5, respectively. In Table 4, it can be observed that most of the molecules do not violate the Lipinski’s rule of five (Pfizer), Ghose, Veber, Egan, and Muegge, except for molecule **4a**, which violates one of Muegge’s rules by having a molecular weight less than 200 g/mol.

In the medicinal chemistry section, several aspects are considered. The PAINS are promiscuous structures that can have biological activity against various molecular targets independently of the pharmacological target, while the Brenk parameters include a list of 105 fragments that could cause toxicity, in addition to being associated with poor pharmacokinetic properties, poor metabolism, and high chemical reactivity. This information guides us to a possible chemical modification with the aim of increasing pharmacological activity and decreasing or avoiding undesired effects. Likewise, a factor of great importance in drug design is the chemical synthesis process; therefore, the prediction of a high success rate in obtaining synthetic compounds is classified as 1 easy and 10 difficult in terms of synthesis. In this sense, it can be observed in Table 4 that the synthesis accessibility scores of the proposed compounds are in a range of 2 to 4, which confirms what was previously reported in that they are relatively easy to obtain [53,54,55,56,57,58].

In summary, in the first screening, which started with 49 compounds, those with an ODLS of 1.0 were selected. Subsequently, with the evaluation of aqueous solubility, pharmacokinetics, and medicinal chemistry, it was possible to choose the compounds that obtained an ODLS equal to or greater than 8.0. To select the compounds that would go on to the next screening, Table 2, Table 3 and Table 4 were compared in order to verify that all of them contained the highest scoring compounds to be evaluated in the next phase of the in silico study, which corresponds to molecular docking.

#### 2.1.2. Binding Mode and Ligand–Protein Interactions by Molecular Docking

**Figure 2:**
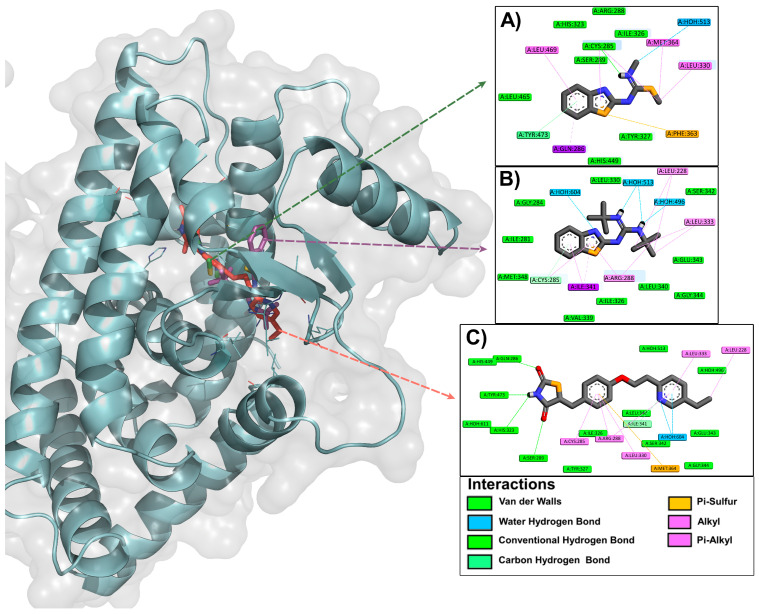
Binding mode and type of interaction presented between ligands **3b** (**A**), **4y** (**B**), and PGZ (**C**) with PPARγ (ID code 2PRG; https://www.rcsb.org/structure/2PRG, accessed on 15 November 2022) [78,79]. Molecular docking analysis was performed by Molegro Virtual Docker Version MVD 2019 7.0 (http://molexus.io/molegro-virtual-docker/, accessed on 1 December 2022) [77], whereas the binding mode and type of interaction were visualized by Drug Discovery Version 20.1.0.19295 (https://www.3ds.com/products/biovia/discovery-studio, accessed on 16 December 2022) [83] and PyMOL Version 3.1 (https://www.pymol.org/, accessed on 22 December 2022) [84].

Molecular docking analysis was performed by Molegro Virtual Docker Version MVD 2019 7.0 (http://molexus.io/molegro-virtual-docker/, accessed on 1 December 2022) [77], evaluating the interactions of each of the ligands selected in the third screening (**3a**, **3b**, **4a**, **4b**, **4c**, **4r**, **4s**, **4x**, and **4y**) with PPARγ protein crystal PDB ID code 2PRG (https://www.rcsb.org/structure/2PRG, accessed on 15 November 2022) [78,79], which was one of the most closely related molecular targets according to the reverse docking previously performed in DIA-DB (https://bio-hpc.ucam.edu/dia-db/index.php, accessed on 6 November 2022) [80]. The ΔG values for the nine best ligands, which were obtained from PRODIGY (https://rascar.science.uu.nl/prodigy/, accessed on 7 December 2022) [81,82], range from −6.4 to −8.4 kcal/mol (Table S5), being among the most promising compounds of the benzothiazolisothiourea and guanidinobenzothiazole series, **3b** (ΔG = −7.8 kcal/mol) and **4y** (ΔG = −8.4 kcal/mol), respectively. It is worth mentioning that, although most of the ΔG values of the evaluated ligands do not exceed the ΔG value of the reference ligand (PGZ, ΔG = −9.5 kcal/mol), most of them present acceptable values (>−7.0 kcal/mol). Figure 2 presents the binding mode and type of interaction, which were visualized by Drug Discovery Version 20.1.0.19295 (https://www.3ds.com/products/biovia/discovery-studio, accessed on 16 December 2022) [83] and PyMOL Version 3.1 (https://www.pymol.org/, accessed on 22 December 2022) [84], between ligands **3b** and **4y** with PPARγ (ID code 2PRG; https://www.rcsb.org/structure/2PRG, accessed on 15 November 2022) [78,79], considering that they were among the best qualified according to their respective ΔG values. However, the interactions of the remaining seven ligands with the protein of interest were also analyzed, finding similar results.

Several studies have identified the forms of interaction of full and partial agonists with PPARγ, which is made up of a Y-shaped ligand-binding domain (LBD) consisting of Arms I, II, and III. Arm I extends towards helix 12 (H12) and is characterized by being polar and widely conserved in the three PPAR subtypes (α, β/δ, and γ). It is linked to the activator function 2 (AF-2) in the C-terminal region that maintains its active conformation through a network of hydrogen bonds with Arm I, which favors ligand binding. Arms II and III are less conserved than Arm I among PPAR subtypes and both are hydrophobic in nature [85,86,87,88]. It has been proposed that several ligands bind to the PPARγ LBD through a hydrophilic interaction with the Arm I region and hydrophobic interactions with regions of Arms II or III. Partial agonists activate PPARγ through a mechanism independent of the H12 conformational change induced by full agonists, since most have been shown to occupy regions of Arms II and III between helix 3 (H3) and the beta sheet, leading to a decrease in H12 stability that affects coactivator binding and, consequently, PPARγ transcriptional activity [85]. Furthermore, the key residues in the LBD that induce receptor activation in the presence of a partial agonist are completely different from those that induce activation in the presence of a full agonist, since almost all partial agonists can stabilize the beta sheet through hydrogen bonding of an acidic group with the amine of the Ser342 backbone. However, partial agonists lacking an acidic group can also stabilize the beta sheet through hydrophobic interactions, especially with the side chain of Ile341. Furthermore, most partial agonists interact hydrophobically with Cys285 of H3 and with Arg288 through electrostatic, hydrophobic, or van der Waals interactions. Partial agonists extending into Arm I of the LBD only form a hydrogen bond with one of the AF2 residues and make electrostatic interactions with other residues in proximity such as Tyr327 or Ser289, although they can also form limited hydrophobic interactions with H12, such as with Leu469. Finally, some partial agonists form other interactions at the edges of the LBD, including pi–pi interactions with Phe282 of helices H3, Phe264 of the loop adjacent to H2′, and Phe363 of helix H7 [85,86,87,88]. Figure 2 shows that the most representative interacting residues are Tyr327, Ser289, Leu469, Cys285, Arg288, Phe282, and Phe363 for compound **3b**, while for **4y** they are Tyr327, Ser289, Cys285, Arg288, Ile341, and Phe363.

In summary, the selection of the best compounds to continue with further studies was carried out based on the score obtained in the prediction of the ADMET properties. However, the ∆G value and the type of ligand–protein interaction obtained from the molecular docking study were decisive in defining the candidates to be evaluated in vivo.

### 2.2. In Vivo Studies

#### 2.2.1. Acute Oral Toxicity (AOT) of Compounds **3b** and **4y**

In Table 5, it can be observed that the administration of compounds **3b** and **4y** did not cause the death of animals at any of the doses evaluated (175, 550, and 1750 mg/kg) based on Guide No. 425 of the Organisation for Economic Co-operation and Development for the evaluation of AOT [89], so the value of the median lethal dose (LD50) is greater than 1750 mg/kg in both cases. These results show that compounds **3b** and **4y** belong to Class IV according to the GHS, indicating that they have low toxicity and thus may be safe for pharmaceutical use [70].

**Figure 3:**
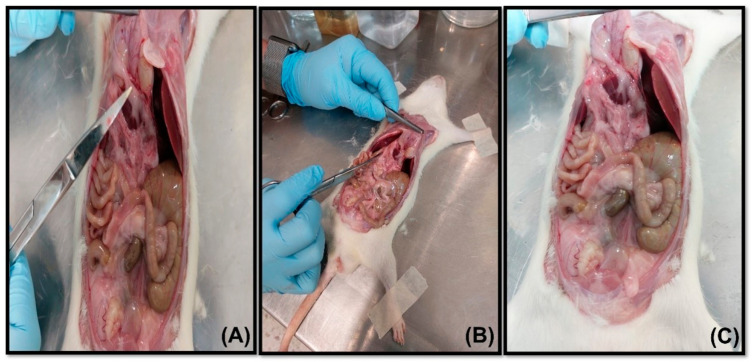
Necropsy performed on animals that were administered vehicle (**A**), compound **3b** (**B**), and compound **4y** (**C**), both at a single dose of 1750 mg/kg.

Figure 3 shows the photographs corresponding to the necropsy performed 15 days after the animals were administered compounds **3b** and **4y**. As can be seen, neither of the two compounds produced obvious macroscopic damage to the organs and tissues of the animals, which presented normal characteristics in terms of size and color (Figure 3B,C) compared to the animal that did not receive treatment and the one that was administered the vehicle (Figure 3A) [87,90,91].

Table 6 presents the values of the organ weights obtained after performing the corresponding necropsy following the acute administration of **3b** and **4y**. As can be seen, there was no significant difference in the weight of any of the organs extracted from the animals treated with both compounds compared to the organs of the animal that was administered with the vehicle. This suggests that **3b** and **4y** do not produce inflammation or other evident metabolic alterations that could have generated a significant increase or decrease in the weight of the organs.

#### 2.2.2. Acute and Subchronic Effect of Compounds **3b** and **4y** in the Rat Model with T2D

Current protocols for the treatment of T2D are initially based on indicating to patients a non-pharmacological treatment consisting of a balanced diet and weight control; however, many of them also require a treatment based on the use of oral hypoglycemic agents to increase insulin sensitivity in adipose tissue, skeletal muscle and liver [5]. Unfortunately, most drugs used to treat T2D have adverse effects. Therefore, the design or discovery of new agents with high antihyperglycemic activity, lipid profile control, and fewer adverse effects becomes of great importance [4,9,87,90,91]. To establish the mechanisms of action of these compounds, various in vivo and in vitro models have been used, which in turn serve as a reference for the evaluation of new chemical entities with possible antidiabetic activity. One of the rat models of T2D can be induced by administration of a single, low dose (35–60 mg/kg) of streptozotocin (STZ), which has been shown to produce hyperglycemia in rats because of its selective cytotoxicity to pancreatic beta cells and minimal toxicity to other organs compared to alloxan [9,87,91,92,93,94,95,96]. The mechanism of action of STZ is related to an increase in the activity of xanthine oxidase and poly (ADP-ribose) polymerase (PARP), which consequently causes apoptotic and necrotic death of pancreatic beta cells. STZ is a nitric oxide (NO) donor, and NO has been shown to induce pancreatic islet cell destruction and mediate the restriction of mitochondrial ATP generation [95]. Despite controversies that exist in using low doses of STZ to induce T2D in rats, a recent study showed that a single intraperitoneal injection of this substance at 50 mg/kg significantly increased plasma glucose levels and insulin resistance at 72 h after its administration, while decreasing serum insulin levels and the sensitivity to this hormone [97]. Furthermore, it has been shown that a low-dose STZ administration mimics the partial loss of β-cell mass in T2D [98]. Therefore, these findings have demonstrated that a single injection of STZ in the range between 45 and 50 mg/kg (considered as low doses) is suitable to induce in rats in a few days a progressive model at the early stage of T2D, showing hyperglycemia and insulin resistance [97,98]. It is well known that insulin resistance in peripheral tissues (skeletal muscle, adipose tissue, and liver) is associated with increased oxidative stress and inflammation in patients with T2D, as well as with the activation of the insulin-signaling pathway through the modulation of the expression and activity of phosphatidylinositol-3-kinase (PI3K) and protein kinase B (PKB, also known as Akt) [87,91,97]. In addition, an increase in the expression and activity of protein kinase C (PKC) has been demonstrated in insulin resistance, which is due to oxidative stress generated from lipoperoxidation and the elevation of free fatty acids, leading to the synthesis of diacylglycerol (DAG) [97]. In this regard, it has been suggested that STZ, by producing reactive oxygen species (ROS) and inflammation, may induce insulin resistance via decreased expression of insulin receptor substrate 1 (IRS-1), PI3K, and PKB/Akt, leading to the impaired translocation of glucose transporter type 4 (GLUT-4) to the plasma membrane in insulin-sensitive tissues. In contrast, an increase in the expression of PKC has been observed [97]. After the administration of animals with a single dose of STZ (45 mg/kg), a significant increase in blood glucose concentration was observed, which ranged from 250 to 350 mg/dL. In addition, one group of animals received a single dose of 5 mg/kg of the drug glibenclamide (GBC), and blood glucose levels were monitored at 0.5, 1, 2, 3, and 4 h (Figure S1). GBC produced a decrease in glucose values, suggesting that functional pancreatic beta cells are still present in the animals [99]. In this regard, GBC is known to be an oral hypoglycemic agent of the secretagogue type, whose mechanism of action involves the blockade of ATP-dependent potassium channels, causing a depolarization of the plasma membrane and calcium gradient signaling, which induces increased insulin release [100]. In addition, to confirm the efficacy of the T2D subchronic model used in the present study, the homeostatic model assessment for insulin resistance (HOMA-IR) index was calculated at the end of treatment (week 4). The results show that in the T2D group, insulin resistance increased compared to the control group (Figure S2C). However, although plasma glucose levels increased significantly in the T2D group compared with the control group (Figure S2A), insulin levels did not change significantly compared with the latter (Figure S2B). This may be explained by the fact that the relationship between insulinemia and insulin resistance in T2D is still not entirely clear. Indeed, it has been demonstrated that in the early stages of disease, the insulin levels do not decrease and/or even increase in animals or patients with diabetes. Therefore, it has been established that insulin levels are elevated in the early stages of T2D, while being in progression may influence insulin resistance [98].

**Figure 4:**
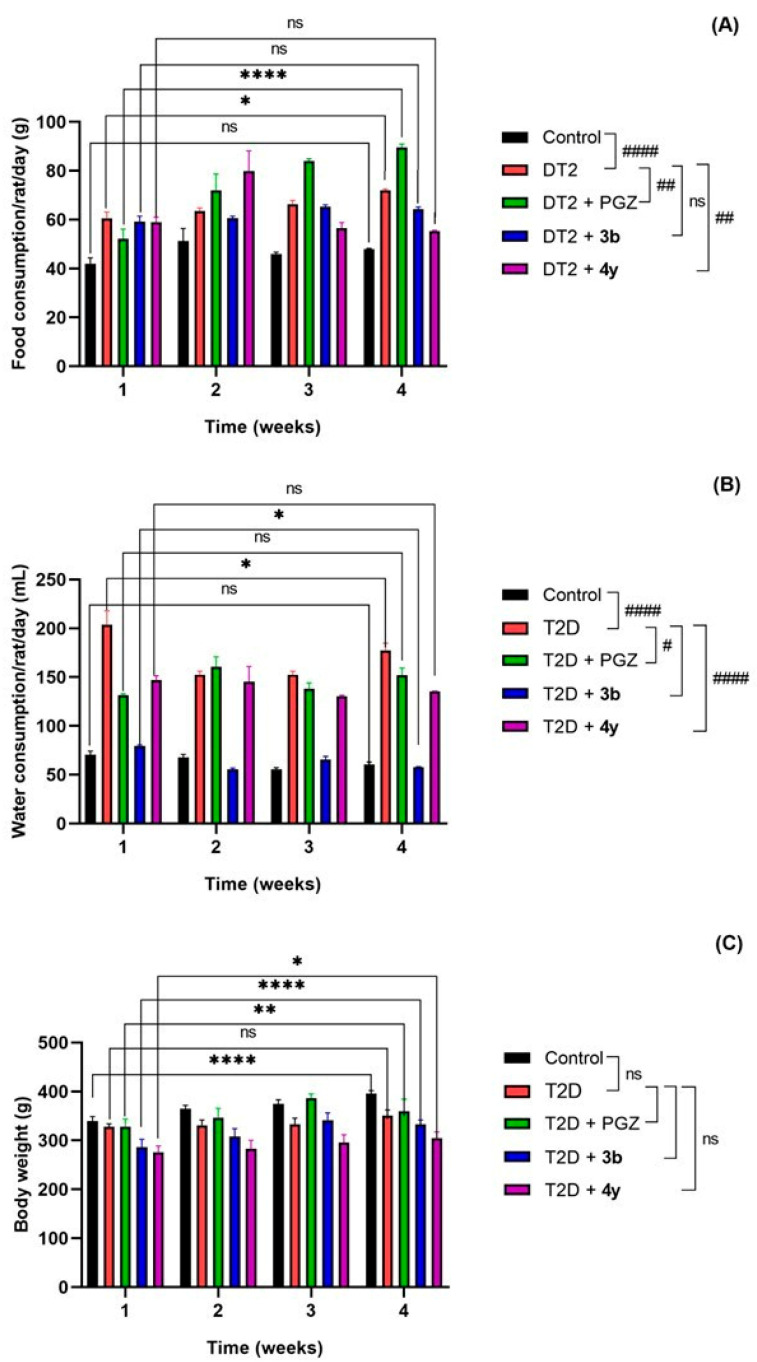
Food consumption (**A**), water consumption (**B**) and body weight (**C**) of T2D animals administered compounds **3b** and **4y** in equimolar ratio to PGZ (15 mg/kg) for 4 weeks. Data are expressed as mean ± SEM and were analyzed by two-way ANOVA with Tukey’s post-hoc test; n = 6. The graphs represent significant and no significant differences (ns) of each treatment group with respect to time (* week 1 vs. week 4) and between them (^#^ control vs. T2D and ^#^ T2D vs. T2D + PGZ, T2D + 3**b**, or T2D + **4y**) at the end of treatment (week 4). * *p*, ^#^ *p* < 0.1; ** *p*, ^##^ *p* < 0.01; **** *p*, ^####^ *p* < 0.0001; ^ns^ *p* > 0.05.

Figure 4A–C shows the food and water consumption, as well as the body weight of the animals with T2D that were administered with compounds **3b** and **4y** in an equimolar ratio to PGZ (15 mg/kg) for 4 weeks. As can be seen in Figure 4A, in the T2D + **3b** and T2D + **4y** groups, food consumption remained constant from the beginning of treatment (week 1) to the end of it (week 4). In the T2D and T2D + PGZ groups, food consumption was significantly higher in week 4 compared to the beginning of treatment (week 1). In Figure 4B it can be observed that water consumption was significantly lower in the T2D + **3b** group in week 4 compared to week 1, while in the T2D + **4y** and T2D + PGZ groups, water consumption remained constant and, in the T2D group, there was a significant decrease in week 4 compared to the start of treatment (week 1).

In Figure 4C, it can be observed that in the T2D + **3b** group, the body weight of the animals increased significantly in week 4 with respect to week 1, and in the T2D + **4y** group, there was also an increase, although this was not significant with respect to time. In the control and T2D + PGZ groups, the weight also increased significantly in week 4 with respect to the beginning of the treatment (week 1), while in the T2D group, the weight remained constant. In this regard, it is well known that PGZ can produce the differentiation of adipose tissue in animals with T2D through the activation of PPARγ, which was demonstrated in this study [87,91]. It is noteworthy that, although the weight of T2D animals administered compounds **3b** and **4y** increased during the treatment period (week 1–4), it was lower compared to that of the group administered PGZ, suggesting that both compounds do not induce adipogenesis compared to this drug.

On the other hand, even though T2D animals consumed food and water constantly and even in greater quantities (in the case of water) than the rest of the experimental groups (T2D + PGZ, T2D + **3b** and T2D + **4y**), their body weight did not change significantly (Figure 4A–C). These results are like the symptoms of T2D patients, which include weight loss despite polyphagia (increased food consumption), polydipsia (increased water consumption), and polyuria (increased urinary volume generation) [87,91]. STZ-induced diabetes is also characterized by weight loss, the decrease in which is due to the degradation of structural proteins such as those that form part of skeletal muscle (cachexia), since it is known that these contribute to weight gain [96]. This was clearly demonstrated in this study, since although there was no significant decrease in the weight of animals with T2D, it was lower compared to that of animals in the control group during the treatment period (week 1–4).

**Figure 5:**
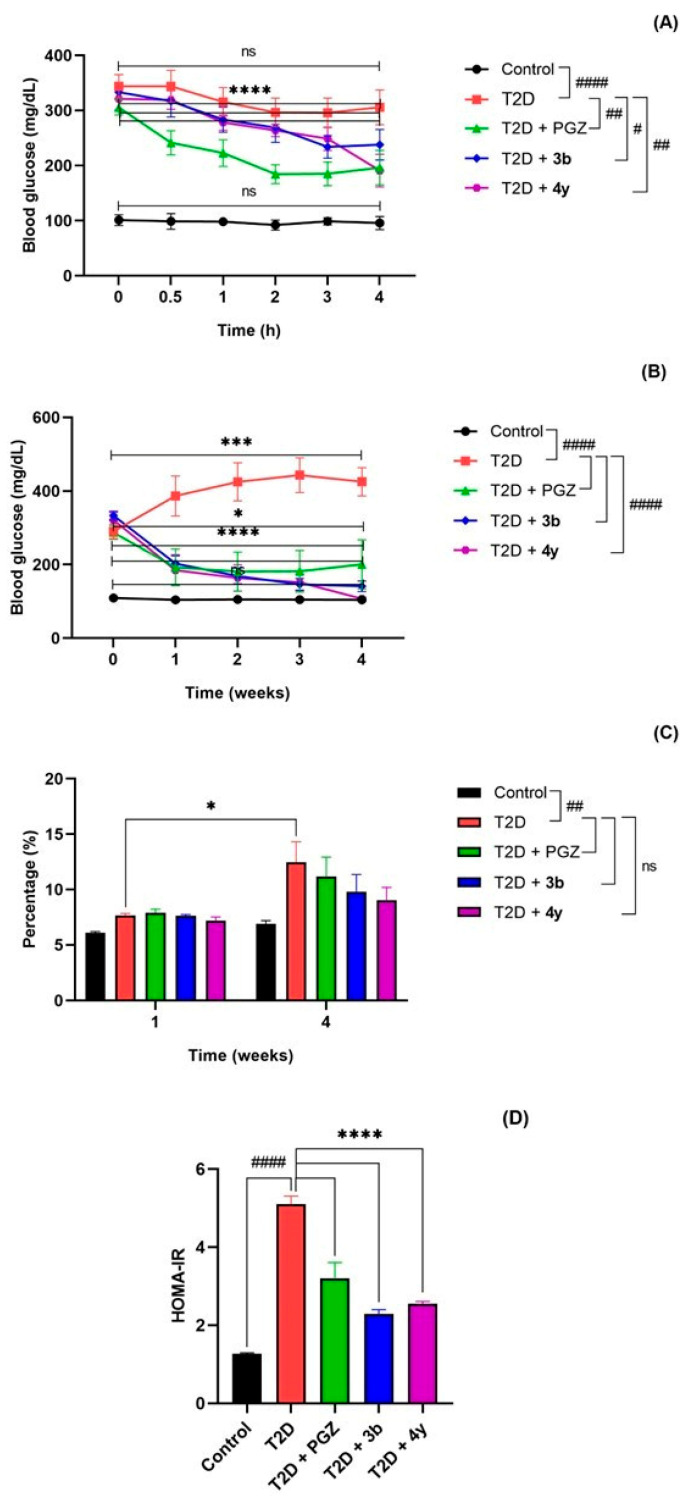
Blood glucose levels in T2D animals administered compounds **3b** and **4y** in equimolar ratio to PGZ (15 mg/kg) as a single dose (**A**), with repeated doses for 4 weeks (**B**), percentage of HbA1c (**C**), and (**D**) HOMA-IR index. Data are expressed as mean ± SEM and were analyzed by two-way ANOVA with Tukey’s post-hoc test; n = 6. The graphs represent significant and no significant (ns) differences of each treatment group with respect to time (* 0 h vs. 4 h or * week 1 vs. week 4) and between them (^#^ control vs. T2D and ^#^ T2D vs. T2D + PGZ, T2D + 3**b** or T2D + **4y**) at the end of treatment (4 h or week 4). * *p*, ^#^ *p* < 0.1, ^##^ *p* < 0.01; *** *p* < 0.001; **** *p*, ^####^ *p* < 0.0001; ^ns^ *p* > 0.05.

Following the acute and subchronic administration of compounds **3b** and **4y** in an equimolar ratio to PGZ (15 mg/kg), blood glucose concentration, HbA1c percentage, and HOMA-IR index were measured (Figure 5A–D). Figure 5A shows that blood glucose levels in animals in the T2D + **3b** group decreased significantly from 1 h to 4 h and in those in the T2D + **4y** group from 2 h to 4 h, both cases with respect to the initial value (0 h). In animals in the TD2 + PGZ group, blood glucose concentration decreased significantly from 0.5 h to 4 h with respect to the start (0 h). In addition, the T2D + **4y** and T2D + PGZ groups showed significant differences with the T2D group and the latter with the control group. It is worth noting that in the T2D + **3b** and T2D + **4y** groups, there was no decrease in blood glucose levels below that of the control group, so these results suggest that, like PGZ, compounds **3b** and **4y** do not cause hypoglycemia in healthy subjects or in those with T2D below normal values [9,87].

Regarding the subchronic administration of **3b** and **4y**, Figure 5B shows that blood glucose concentration decreased significantly in both groups from week 1 until the end of treatment (week 4), as in the T2D + PGZ group. However, as can be seen in the graph, unlike the T2D + **3y** and T2D + **4y** groups, blood glucose values in the T2D + PGZ group did not reach normal levels (<100 mg/dL) at week 4, which is consistent with what has been reported in previous studies [9,87,91,101]. HbA1c is a sensitive marker used to detect early diabetes in high-risk individuals [96]. Figure 5C shows that the percentage of HbA1c in the T2D + **3b**, T2D + **4y**, and T2D + PGZ groups decreased at week 4 from baseline (week 1). Furthermore, the percentage of HbA1c also decreased in the three groups compared to the T2D group, but without reaching the values of the control group at week 4 of treatment. In this regard, it has been shown that the percentage of HbA1c strongly correlates with fasting blood glucose concentration in patients with diabetes. The glycation of hemoglobin is irreversible during the 120 days of life of red blood cells in the body. The assessment of changes in blood glucose levels is cumulative over a period of 4 to 8 weeks. The percentage of HbA1c is stable, does not vary during the day, and does not depend on recent changes in diet. Hemoglobin has free access to glucose, which passively diffuses through the red blood cell membrane.

However, the glycation mechanism increases due to glycemic stress caused by the metabolic disturbance produced by the diabetic state [102]. In the present study, a significant increase in HbA1c levels was observed in addition to hyperglycemia in rats with T2D compared to healthy controls, indicating their poor glycemic status, as previously reported [96,102]. As expected, the metabolic alteration decreased with the administration of compounds **3b** and **4y**, as well as with PGZ. Furthermore, to demonstrate that compounds **3b** and **4y** are capable of decreasing insulin resistance, the HOMA-IR index was calculated. In Figure 5D, it is possible to observe that in the T2D + **3b**, T2D + **4y** and T2D + PGZ groups, there is a significant decrease in insulin resistance with respect to the T2D group, which suggests that **3b** and **4y** may act in a similar way to PGZ, that is, activating PPARγ, which in turn leads to the modulation of GLUT-4 expression and its consequent translocation to the plasma membrane, favoring insulin sensitivity and glucose uptake in peripheral tissues [87,91,97].

Regarding the lipid profile, it can be observed in Table 7 that in animals with T2D administered with compounds **3b** and **4y**, the concentrations of triglycerides (TGs), total cholesterol (T-Cho), and low-density lipoproteins (LDL-Cs) decreased with respect to the T2D group, while high-density lipoproteins (HDL-Cs) increased (week 4). It is worth mentioning that these results are comparable with those of the T2D + PGZ group, the effect of this drug on the lipid profile being well demonstrated in previous studies [9,87,91,96,101].

Oxidative stress may also play a central role in the pathogenesis of diabetic complications such as impaired glucose and lipid metabolism that promote hyperglycemia and dyslipidemia through the overproduction of reactive oxygen species (ROS). These complications are associated with the development of atherosclerosis and cardiovascular diseases. Hyperglycemia is accompanied by elevated levels of TG, T-Cho, and LDL-C and decreased HDL-C levels in diabetic rats, in which the abnormally high serum lipid concentrations are mainly due to the increased mobilization of free fatty acids from fat stores. Insulin activates the enzyme lipoprotein lipase (LPL) that hydrolyzes TG under normal conditions; however, it is not activated in the diabetic state due to insulin deficiency, resulting in hypertriglyceridemia. Circulating LDL-C is recaptured in the liver through specific receptors that clear the circulation. The increased serum LDL-C concentration in diabetic rats could be due to a defect in the LDL-C receptor, either through a failure in its production or function. ROS can stimulate the oxidation of LDL-C, which can be taken up by scavenger receptors in macrophages, leading to the formation of foam cells and atherosclerotic plaques. HDL-C is a cardioprotective lipid by reversing T-Cho transport, thus neutralizing the atherogenic effect of oxidized LDL-C, preventing coronary heart disease. The greater decrease in HDL-C could be due to the greater increase in LDL-C and VLDL-C since there is a reciprocal relationship between VLDL-C and HDL-C concentration. The decreased activity of the enzyme lecithin cholesterol acyltransferase (LCAT) could be responsible for the decreased activity of HDL-C [96,101]. In this study, animals with T2D administered compounds **3b** and **4y** showed an increase in HDL-C and a reduction in TG, T-Cho, and LDL-C, so these results confirm that both compounds, like PGZ, can reduce the risk of cardiovascular diseases by restoring the unbalanced lipid profile.

In Table 8, it can be observed that the administration of compounds **3b** and **4y** to animals with T2D did not produce a significant change in the serum concentrations of the enzymes alanine aminotransferase (ALT/GPT), aspartate aminotransferase (AST/GOT), and gamma-glutamyl transferase (GGT) with respect to the control group.

These results are like those observed in the T2D + PGZ group, as well as with previous studies in which it has been shown that this drug does not produce an increase in the catalytic activity of liver enzymes in both rats and humans with T2D [87,101]. Furthermore, since these results correlate with those obtained in the in silico study, as well as with the macroscopic analysis performed after subchronic treatment with **3b** and **4y**, in which it was shown that both compounds do not cause tissue damage, particularly at the hepatic level, it is suggested that they are safe and do not produce harmful effects on the liver. On the contrary, in animals administered with PGZ there was an increase in adipose tissue, which is well known to be one of the adverse effects of TZDs [9,87,91,101].

In summary, compounds **3b** and **4y** administered to animals with T2D favor the decrease in blood glucose concentration and improve dyslipidemia, which could be explained based on a mechanism of action like that of PGZ that improves insulin sensitivity throughout the body, instead of stimulating its secretion from pancreatic beta cells. In addition, the attenuating effect on hyperlipidemia (although not significant) could result either from the inhibition of TG synthesis in the liver or from the increase in its clearance in the periphery by stimulating the LPL enzyme, and/or the inhibition of intestinal absorption of dietary T-Cho.

## 3. Materials and Methods

### 3.1. Chemicals

All chemical reagents and solvents were of standard analytical grade and used without further purification (Sigma Aldrich; Toluca, State of Mexico, Mexico). For the quantification of metabolic (glucose, HbA1c, TG, T-Cho, HDL-C and LDL-C) and hepatic parameters (ALT/GPT, AST/GOT and GGT) solid-phase test strips with a coded chip (SPOTCHEM Ii Glu, Catalog Number: 77160; SPOTCHEM Ii KENSHIN-2, Catalog Number: 77188, Arkray; Kyoto, Japan) were used.

### 3.2. In Silico Studies

#### 3.2.1. Prediction of ADMET Properties

The 49-compound library was generated from two different series containing the 2-aminobenzothiazole nucleus linked to isothioureas (**3a**–**w**) or guanidines (**4a**–**z**). The chemical structures were drawn, and the Simplified Molecular Input Line Entry System (SMILES) code of each compound was obtained from the Molinspiration Cheminformatics online server (https://www.molinspiration.com/, accessed on 21 September 2022) [59]. The physicochemical properties obtained from the latter are molecular weight (MW), octanol–water partition coefficient (cLogP), number of hydrogen bond acceptors (nON) and donors (nOHNH), and number of rotatable bonds (nrotb) and topological polar surface area (TPSA), among others. On the other hand, with the DataWarrior (https://openmolecules.org/datawarrior/, accessed on 25 September 2022) [60,61], in addition to obtaining some of the aforementioned properties (PM, cLogP, ASPT, etc.), the prediction of the toxicity risk of the compounds with respect to mutagenicity (M), tumorigenicity (T), irritant effects (IE) and reproductive effects (RE) was made. These theoretical characteristics were considered to assign an overall drug-likeness score (ODLS) to the evaluated compounds to select the best ones, that is, those that comply with Lipinski’s rule of five [62,63] and the recommendations of Veber et al. [64]. Subsequently, the prediction of aqueous solubility (logS), pharmacokinetic properties (absorption, metabolism, and toxicity), and medicinal chemistry of the compounds selected from the previous virtual screening was obtained using the SMILES code on the online servers ProTox 3.0 (https://tox.charite.de/protox3/, accessed on 5 October 2022) [68,69] and SwissADME (http://www.swissadme.ch/, accessed on 12 October 2022) [71,72].

#### 3.2.2. Analysis of the Binding Mode and Ligand–Protein Interactions by Molecular Docking

In order to establish whether one of the molecular targets of interest in diabetes that was more closely related to the proposed ligands is PPARγ, a reverse molecular docking study was first performed using the online server DIA-DB (https://bio-hpc.ucam.edu/dia-db/index.php, accessed on 6 November 2022) [80] and the reference drug PGZ, considering the association of its mechanism of action with that of other previously reported benzothiazole derivatives [24,40,41,42,43,46,47]. Therefore, a conventional (simple), rigid, and directed molecular docking study was performed. Two-dimensional (2D) structures of the proposed ligands were drawn and pre-optimized using ChemSketch, Version 2022.1.2 (https://www.acdlabs.com/products/draw_nom/draw/chemsketch/, accessed on 10 November 2022) [103] and then saved in *.mol format. Subsequently, the integrity of the ligands was verified, the structures were energetically minimized using a MMFF molecular mechanics method in Spartan Student Edition Version 8 (8.0.6) (https://www.wavefun.com/, accessed on 12 November 2022) [104,105], and the generated file was saved in *.spartan format. All torsions were allowed for rotation during molecular docking and the generated *.mol file was converted to *.pdb format with Spartan Student Edition Version 8 (8.0.6) (https://www.wavefun.com/, accessed on 12 November 2022) [104,105]. The crystal of the protein of interest was chosen from the RCSB PDB database (https://www.rcsb.org/, accessed on 15 November 2022) [106,107], from which the third-dimensional (3D) structure and PDB conformers of it were obtained. Specifically, the PPARγ crystal chosen was the one with ID code 2PRG (https://www.rcsb.org/structure/2PRG/, accessed on 15 November 2022) [78,79].

Subsequently, to carry out the molecular docking studies between the ligands and the protein, the Molegro Virtual Docker Version MVD 2019 7.0 (http://molexus.io/molegro-virtual-docker/, accessed on 1 December 2022) [77] was used. First, the method was validated, with the selection criterion being a root mean square deviation (RMSD) value < 2 Å. For the PPARγ protein crystal PDB ID code 2PRG (https://www.rcsb.org/structure/2PRG/, accessed on 15 November 2022) [78,79], the coordinates were X = 49.31, Y = −37.06, and Z = 19.13, with a radius of 10 Å, using 3 search algorithms (MoldDock Optimaizer, MoldDock SE and Iterated simplex) and 4 scoring functions (MoldDock Score, MoldDock Score GRID, PLANTS Score and PLANTS Score GRID). Ten runs were performed with the selected method (MoldDock Optimaizer search algorithm and PLANT Score scoring function), with 1500 maximum iterations in a population size of 50. Once the method was validated with the co-crystallized PPARγ ligand (PDB ID code 2PRG; https://www.rcsb.org/structure/2PRG/, accessed on 15 November 2022) [78,79], the reference ligand PGZ was subjected to the previously established conditions to determine its binding mode and affinity for said protein. In addition, the dominant microspecies at physiological pH (7.4) were searched for using the MarvinSketch Version 19.21.7 program, and the necessary modifications were made before starting the molecular docking with each of the ligands to be evaluated. At the end of this procedure, the generated files of each ligand were exported to a format with *.pdb extension for the subsequent analysis of the ligand–protein interactions by using Drug Discovery Version 20.1.0.19295 (https://www.3ds.com/products/biovia/discovery-studio/, accessed on 16 December 2022) [83] and PyMOL Version 3.1 (https://www.pymol.org/, accessed on 22 December 2022) [84] that allow for the visualization of the interactions in 2D and 3D, respectively. In addition, the ∆G value of each of the ligand–protein interactions was calculated using PRODIGY (https://rascar.science.uu.nl/prodigy/, accessed on 7 December 2022) [81,82].

### 3.3. Synthesis and Structural Identification of Compounds ***3b*** and ***4y***

The synthesis and structural identification of compounds **3b** and **4y** was carried out based on previously reported data with slight modifications [53,55,57]. The respective spectra (^1^H and ^13^C NMR, IR, and MS) and chromatograms (HPLC) are included in the Supplementary Materials (Figures S3–S12).

#### 3.3.1. Intermediate Compound Dimethyl Benzo[d]thiazol-2-ylcarbonimidodithioate (**2**)

**Scheme 1:**
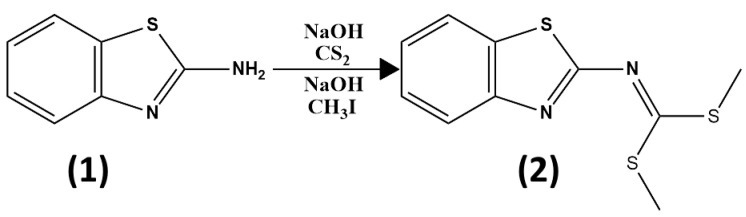
General synthesis route of the intermediate compound dimethyl benzo[d]thiazol-2-ylcarbonimidodithioate (**2**) from 2-aminobenzothiazole (**1**).

The synthesis of compound **2** was carried out based on previously reported [53,55,57]. First, 7.51 g (1 equivalent: 0.05 mol) of 2-aminobenzothiazole (**1**) was added to 50 mL of N,N-dimethylformamide (DMF), and the resulting solution was placed on an ice bath and stirring was started. Then, 3 mL (1 equivalent) of a 20 M sodium hydroxide (NaOH) solution was added drop-wise to the reaction mixture. When the formation of a gray precipitate was observed, stirring was maintained for 30 min more and, subsequently, 6 mL (1.2 equivalents: 0.1 mol) of carbon disulfide (CS2) was added drop by drop, leaving the reaction mixture stirring for another 30 min. Subsequently, other 3 mL (1 equivalent) of the 20 M NaOH solution was added and the reaction was kept stirring for 60 min. Then, 5.7 mL (2.2 equivalents: 0.1 mol) of methyl iodide (MeI) was added and after approximately 5 min, the formation of a bright yellow precipitate was observed. After 2 h of stirring on the ice bath, 900 mL of cold distilled water was added, and the reaction was stopped (Scheme 1). The precipitate was filtered and washed several times with cold water and the solid obtained was recrystallized from a mixture of ethanol/water in an undefined proportion. The product yield was 80%.

#### 3.3.2. Compound Methyl (E)-N′-(Benzo[d]thiazol-2-yl)-N-methylcarbamimidothioate (**3b**)

**Scheme 2:**
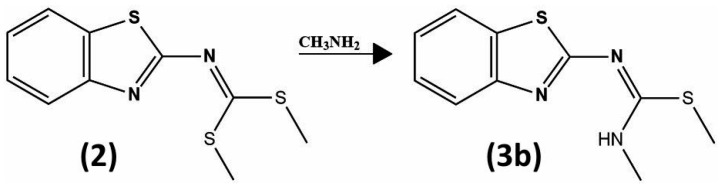
General synthesis route of methyl (E)-N′-(benzo[d]thiazol-2-yl)-N-methylcarbamimidothioate (**3b**) from the intermediate dimethyl benzo[d]thiazol-2-ylcarbonimidodithioate (**2**).

The synthesis of compound **3b** was carried out based on previously reported [53,55,57]. First, 1 g (1 equivalent: 0.0039 mol) of the compound dimethyl benzo[d]thiazol-2-ylcarbonimidodithioate (**2**) was added to 10 mL of ethanol, and stirring was started. Then, 0.31 mL (1 equivalent: 0.0039 mol) of methylamine was added, and the reaction mixture was left stirring at room temperature for 48 h (Scheme 2). After the time elapsed, the solvent was evaporated, allowing for the precipitation of the product. The mobile phase to monitor the reaction by thin-layer chromatography was composed of ethyl acetate, n-hexane and chloroform (ratio 1:20:1). The purification of the product was carried out by fractional recrystallization with enough hot ethanol to dissolve the yellow solids formed.

#### 3.3.3. Compound 2-(Benzo[d]thiazol-2-yl)-1,3-di-tert-butylguanidine (**4y**)

**Scheme 3:**
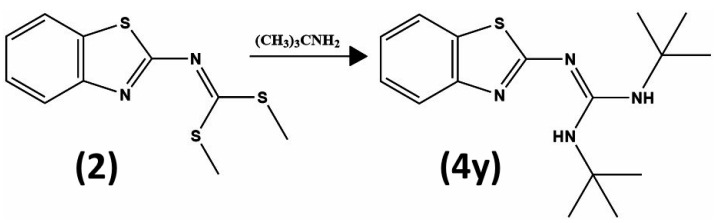
General synthesis route of 2-(benzo[d]thiazol-2-yl)-1,3-di-tert-butylguanidine (**4y**) from the intermediate dimethyl benzo[d]thiazol-2-ylcarbonimidodithioate (**2**).

The synthesis of compound **4y** was carried out based on previously reported [53,55,57]. 1 g (0.0039 mol) of the compound dimethyl benzo[d]thiazol-2-ylcarbonimidodithioate (**2**) in 20 mL of ethanol was added and stirring was started. 0.84 mL (2 equivalents: 0.008 mol) of tert-butylamine was added and the reaction mixture was left to reflux and stir for 8 days, adding 0.1 mL of the mentioned amine every 24 h (Scheme 3). After the time elapsed, the solvent was evaporated allowing the precipitation of the product. The mobile phase to monitor the reaction by thin-layer chromatography was composed of ethyl acetate, n-hexane, and acetone (ratio 3:35:2). The product was purified by silica gel column chromatography (G 60 F254), using the same eluent system as for thin-layer chromatography. The content of each fraction was monitored using a reference sample of the compound, which was previously obtained by fractional recrystallization.

#### 3.3.4. Structural Identification

The purity of the products was determined by high-performance liquid chromatography (HPLC), while the melting point was measured in triplicate for each compound, obtaining an average of the respective temperatures. The identification of the chemical structures was performed by hydrogen-1 and carbon-13 nuclear magnetic resonance spectroscopy (^1^H and ^13^C NMR), the spectra were obtained from an equipment at 300.08 MHz and 74.46 MHz, respectively, using deuterated chloroform and tetramethylsilane as internal standard. The infrared (IR) spectra were recorded on Fourier transform equipment using a zinc selenide film. Finally, the presence of the molecular ion of each compound was corroborated in a mass spectrometer by using the electrospray ionization mode (ESI). Once compounds **3b** and **4y** were identified, solubility tests were carried out on each of them for evaluation in biological assays.

**3b**: yellowish crystals, purity 97.18%; mp 72 °C; IR (Kbr) Vmax 1312, 1444, 1555, 2900, 3275 cm^−1^; MS (micrOTOF-Q): [M+H]+ calc. for C_10_H_11_N_3_S_2_: 238.04 found: 238.0467; ^1^H- and ^13^C-NMR (300 MHz, CDCl_3_) (Figures S3–S12).

**4y**: whitish crystals, purity 97.11%; mp 87 °C; IR (Kbr) Vmax 1200, 1610, 3450 cm^−1^; MS (micrOTOF-Q): [M+H]+ calc. for C_16_H_24_N_4_S: 305.17 found: 305.1794; ^1^H- and ^13^C-NMR (300 MHz, CDCl_3_) (Figures S3–S12).

### 3.4. In Vivo Studies

#### 3.4.1. Animals

Male Wistar rats weighing 200 ± 20 g were acquired from the Centro de Investigación y de Estudios Avanzados (CINVESTAV) del Instituto Politécnico Nacional (IPN) Unidad Zacatenco. They were housed in polypropylene cages under controlled temperature conditions (20–25 °C) and light/dark cycles of 12 × 12 h, with food (standard) and water ad libitum. Before performing the experiments, the animals were adapted to their new habitat during a one-week acclimatization period. All animals were handled and euthanized in accordance with humane endpoint considerations [108,109].

The study was conducted in accordance with the guidelines of the Declaration of Helsinki and based on the ARRIVE Essential 10 guidelines for the protocol for the use of laboratory animals [110]. The protocol was approved by the Institutional Research Committee on the Care and Use of Laboratory Animals (ESM-CICUAL-02/20-03-2013) of the Escuela Superior de Medicina (ESM) of the Instituto Politécnico Nacional (IPN), Mexico City, Mexico. It complies with the Mexican norm for this matter (NOM-062-ZOO-1999, Technical Specifications for the Production, Care, and Use of Laboratory Animals, SAGARPA), as well as the Guide for the Care and Use of Laboratory Animals of the National Research Council and National Institutes of Health (NIH Publications No. 8023, revised 1978).

#### 3.4.2. Experimental Design

Animals were randomly assigned for all experiments [110]. Specifically, group organization for the evaluation of compounds **3b** and **4y** in the T2D model was carried out as described in Table 9.

#### 3.4.3. Acute Oral Toxicity (AOT) Test of Compounds **3b** and **4y**

The LD50 value of compounds **3b** and **4y** was estimated using the Up-and-Down method of OECD Guideline 425 [89]. According to the latter, a male Wistar rat (200 ± 20 g) was administered a dose of 175 mg/kg and was observed continuously for 48 h. As the animal survived, another animal received an increasing dose of 550 mg/kg (progression factor 3.2). As there were no toxic effects in the second rat, a third animal received an increasing dose of 1750 mg/kg and as it did not die either, two others were administered the same dose. All animals were kept under observation for 14 days, and 24 h later they were taken to the humane endpoint for macroscopic examination of organs and tissues. Finally, the LD50 value of the compounds was estimated to determine their degree of toxicity and classified based on the GHS [70].

#### 3.4.4. Evaluation of Acute and Subchronic Effect of Compounds **3b** and **4y** in the Rat Model with T2D

One week after the acclimatization period (week 0), animals were fasted for 12 h and then administered a single dose of 45 mg/kg STZ dissolved in 0.1 M citrate buffer pH 4.5 intraperitoneally (i.p.). Whole-blood glucose levels were measured with a glucometer (FreeStyle, Optium Neo) and corresponding strips (FreeStyle, Optium) every third day for 1 week, with samples obtained by venipuncture of the rat’s tail. Animals that maintained a blood glucose level between 250 and 350 mg/dL were considered for the study [9,87,91,92,93,94,95,96]. In order to corroborate that the generated model was closer to the pathophysiological characteristics of T2D, the animals with hyperglycemia were administered the twelfth day after the administration of STZ (week 2) with the drug GBC (secretagogue) at a dose of 5 mg/kg, and the changes in blood glucose levels were measured at 0.5, 1, 2, 3, and 4 h, waiting for them to decrease [99]. In addition, the homeostasis model assessment of insulin resistance (HOMA-IR) was used to assess β-cell function and insulin resistance by quantifying basal glucose and insulin at the end of treatment (week 4). Insulin levels were quantified using the Human insulin (INS) ELISA Kit (MyBioSource, Catalog Number: MBS704195, San Diego, CA, USA). Then, the HOMA-IR index was calculated as follows: HOMA-IR = fasting glucose (mmol/L) × fasting insulin (μU/mL)/22.5 [97,98].

In the second week after STZ administration and induction of the T2D model, animals were administered orally (p.o.) with compounds **3b** or **4y** at an equimolar dose relative to the positive control (PGZ, 15 mg/kg; Aurax^®^, Farmacia San Pablo, CDMX, Mexico) [9,87,91,101]. For the acute evaluation, blood glucose concentration was first measured (time 0) in the animals; subsequently, they received a single dose of PGZ, and then blood glucose concentration was measured again at 0.5, 1, 2, 3, and 4 h to observe the effect of the treatment administered in the animals. In the case of subchronic evaluation, compounds **3b** or **4y** were administered daily at the previously mentioned dose (equimolar ratio with PGZ, 15 mg/kg), and blood glucose levels were monitored every 7 days until the end of treatment in the fourth week after its initiation. In addition, quantification of glucose, HbA1c, TG, T-Cho, HDL-C, and LDL-C, as well as the catalytic activity of the enzymes ALT/GPT, AST/GOT, and GGT were performed at the end of treatment to demonstrate its effectiveness. For this purpose, blood samples were obtained by venipuncture of the rat’s tail and, only in the case of HbA1c, it was immediately measured from a whole-blood sample using a portable analyzer (BioHermes, GluCoA1c, Wuxi, China) and test kit (BioHermes, GluCoA1c Glycohemoglobin Test Kit), while for the determination of the other parameters, the blood samples were kept at room temperature for 30 min and then centrifuged at 4000 RPM for 15 min at 4 °C. Finally, the serum was stored at −8 °C until it was measured in an automated dry chemistry analyzer (Arkray, SPOTCHEM EZ, Fushimi-ku, Kyoto, Japan), and strips were used for the quantification of the parameters (SPOTCHEM Ii Glu, Catalog Number: 77160, Glu; SPOTCHEM li KENSHIN-2, Catalog Number: 77180, GPT, GOT, GGT, TG, T-Cho, HDL-C), for a maximum period of 2 days [101].

Food and water consumption were monitored every third day. The animals’ body weight was also recorded from the day of their arrival at their new accommodation and then every 7 days throughout the study [9,87,91].

### 3.5. Ex Vivo Studies

#### Sample Collection and Processing

At the end of the treatments (week 4), the animals were fasted for 12 h and the following day the last blood samples were obtained (following the previously described procedure) before being taken to the humane endpoint. Once the animals were sacrificed, a macroscopic examination of the organs and tissues was performed to rule out signs of toxicity induced by the subchronic administration of compounds **3b** or **4y** [9,87,91,101].

### 3.6. Statistical Analysis

Data obtained in the in vivo studies are expressed as mean ± standard error of the mean (SEM) with n = 6. For all parameters, comparisons between groups were carried out using a two-way analysis of variance (ANOVA) followed by Tukey’s multiple-comparisons test. All statistical tests were performed and graphed using GraphPad software, version 8.2.1, where a *p*-value < 0.05 was considered to indicate whether there was a significant difference in each group with respect to time and between each treatment.

## 4. Conclusions

Compounds **3a**, **3b**, **4a**, **4b**, **4c**, **4r**, **4s**, **4x**, and **4y** selected from the in silico study could be useful for the treatment of T2D and its micro- and macrovascular complications, based on their ADMET properties, as well as the ΔG values obtained with the PPARγ protein. In this study, compounds **3b** and **4y** were evaluated in the T2D rat model, which have an antihyperglycemic and hypolipidemic effect, in addition to not causing liver damage, compared with the reference drug PGZ. Therefore, it is suggested that the mechanism of action of both compounds could be associated with PPARγ agonism, although it is not ruled out that **3b** and **4y** may also act on other molecular targets, thus preventing micro- and macrovascular complications of T2D. However, further preclinical trials are needed to clearly demonstrate the antidiabetic effect of both compounds before they can be evaluated in the clinical phase.

## Figures and Tables

**Table 1:** Benzothiazolisothioureas (**3**) and guanidinobenzothiazoles (**4**) with the highest score obtained from the values of their physicochemical, pharmacological, and toxicological properties by online servers.

Compound	Chemical Structure
**3a**	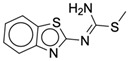
**3b**	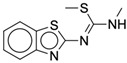
**4a**	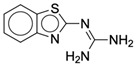
**4b**	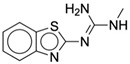
**4c**	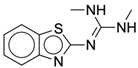
**4r**	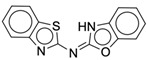
**4s**	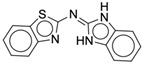
**4x**	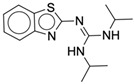
**4y**	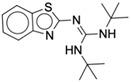

Abbreviations: **3a**: methyl *N*’-(1,3-benzothiazol-2-yl)carbamimidothioate; **3b**: methyl (E)-N′-(benzo[d]thiazol-2-yl)-N-methylcarbamimidothioate; **4a**: N″-(1,3-benzothiazol-2-yl)guanidine; **4b**: 2-(benzo[d]thiazol-2-yl)-1-methylguanidine; **4c**: 2-(benzo[d]thiazol-2-yl)-1,3-dimethylguanidine; **4r**: N-(benzo[d]thiazol-2-yl)benzo[d]oxazol-2(3H)-imine; **4s**: N-(benzo[d]thiazol-2-yl)-1,3-dihydro-2H-benzo[d]imidazol-2-imine; **4x**: 2-(benzo[d]thiazol-2-yl)-1,3-diisopropylguanidine; **4y**: 2-(benzo[d]thiazol-2-yl)-1,3-di-tert-butylguanidine.

**Table 2:** Physicochemical properties and toxicity parameters of the best benzothiazolisothioureas (**3**) and guanidinobenzothiazoles (**4**) obtained from Molinspiration Cheminformatics (https://www.molinspiration.com/, accessed on 21 September 2022) [59] and DataWarrior (https://openmolecules.org/datawarrior/, accessed on 25 September 2022) [60,61].

Compound	Physicochemical Properties						Toxicity (DW)				ODLS
	MW (g/mol)	cLogP	nOHNH	nON	nrotb	TPSA (Å^2^)	M	T	IE	RE	
**3a**	223.33	2.41	2	3	2	51.28	X	X	X	X	1.0
**3b**	237.35	2.79	1	3	3	37.28	X	X	X	X	1.0
**4a**	192.25	2.29	4	4	1	77.30	X	X	X	X	1.0
**4b**	206.27	2.67	3	4	2	63.31	X	X	X	X	1.0
**4c**	220.30	3.04	2	4	3	49.31	X	X	X	X	1.0
**4r**	267.31	4.14	1	4	1	54.19	X	X	X	X	1.0
**4s**	266.33	4.04	2	4	1	56.84	X	X	X	X	1.0
**4x**	276.41	4.38	2	4	5	49.31	X	X	X	X	1.0
**4y**	304.46	5.41	2	4	5	49.31	X	X	X	X	0.92
PGZ	356.45	3.07	1	5	7	68.30	X	X	X	X	1.0

Abbreviations and definitions: **PGZ:** pioglitazone. **DW:** DataWarrior. **MW:** molecular weight (“relative” molecular mass (Mr), which is the ratio of the mass of a molecule to one-twelfth of the mass of carbon 12). **cLogP:** octanol–water partition coefficient (distribution of an organic compound between octanol and water phases, quantifying the substance’s lipophilic and hydrophilic properties). **nOHNH** and **nON:** number of hydrogen bond donors and acceptors, respectively (specific type of electrostatic interaction between a proton attached to an electronegative atom (such as N or O), and a lone pair of electrons on an electronegative atom such as N, O, or F. The former is called the H-bond donor, the latter the H-bond acceptor). **nrotb:** number of rotatable bonds (movement around a metal-to-ligand bond, allowing for increased conformational flexibility in the construction of molecular assemblies with predefined shapes in coordination chemistry). **TPSA:** topological polar surface area (surface associated with heteroatoms and polar hydrogen atoms in a molecule, excluding nonpolar elements like carbon and halogens. It is a sensitive parameter influenced by the 3D conformation of the molecule and is often calculated using various methods such as dynamic averages and fragment-based increments). **M:** mutagenicity (aspect of genotoxicity manifested by mutations, i.e., heritable structural and/or numerical alterations in the DNA that irreversibly and permanently alter its information content). **T:** tumorigenicity (cancer is abnormal cell division or uncontrolled growth in response to various tumorigenic factors). **IE:** irritant effect (related to any non-corrosive substance that on immediate, prolonged, or repeated contact with normal living tissue produces a local inflammatory reaction). **RE:** reproductive effect (adverse effect on any aspect of male or female reproductive structures or functions, on the developing offspring, or on lactation, which would interfere with the development of normal offspring through sexual maturity, in turn capable of normal reproduction). **X**: it does not present the effect. **ODLS:** overall drug-likeness score (sum of the molecular physicochemical properties that are characteristic of chemicals known as drugs. Indeed, drug-likeness is often used to describe pharmacokinetic and safety and can also be understood as compounds with desirable ADMET properties). The definitions of physicochemical properties and toxicity parameters were obtained from https://www.sciencedirect.com/topics/ (accessed on 25 September 2022).

**Table 5:** Data from the AOT test performed with compounds **3b** and **4y**.

Compound 3b			
Doses (mg/kg)	Mortality Rate (%)	LD50 (mg/kg)	GHS Category
175	0	>1750	Class IV
550	0		
1750	0		
**Compound 4y**			
**Doses (mg/kg)**	**Mortality Rate (%)**	**LD50 (mg/kg)**	**GHS Category**
175	0	>1750	Class IV
550	0		
1750	0		

Abbreviations: LD50: median lethal dose; GHS: Globally Harmonized System of Classification and Labeling of Chemicals [70].

**Table 6:** Weight of organs obtained from the necropsy performed 15 days after administration of compounds **3b** and **4y**.

Compound	Organ Weight (g)				
	Spleen	Stomach	Liver	Intestine	Kidney
Vehicle	0.90	3.41	14.08	26.23	2.85
3b *	0.82 ± 0.05	2.93 ± 0.49	13.71 ± 2.11	25.04 ± 1.04	2.50 ± 0.04
4y *	0.84 ± 0.04	2.37 ± 0.40	14.71 ± 2.11	24.04 ± 0.94	2.95 ± 0.05

* Compounds **3b** and **4y** were administered at a single dose of 1750 mg/kg.

**Table 7:** Lipid profile of animals with T2D administered compounds 3b and 4y in equimolar ratio to PGZ (15 mg/kg) for 4 weeks.

Group	Parameter			
	TG (mg/dL)	T-Cho (mg/dL)	HDL-C (mg/dL)	LDL-C (mg/dL)
Control	107 ± 8	85 ± 3	10 ± 0	83 ± 3
T2D	128 ± 29	95 ± 11	12 ± 1	92 ± 10
T2D + PGZ	65 ± 14	79 ± 4	14 ± 2	76 ± 4
T2D + **3b**	91 ± 12	79 ± 3	13 ± 1	76 ± 2
T2D + **4y**	71 ± 15	74 ± 3	14 ± 2	71 ± 3

Abbreviations: T2D: type 2 diabetes; PGZ: pioglitazone; TG: triglycerides; T-Cho: total cholesterol; HDL-C: high-density lipoprotein; LDL-C: low-density lipoprotein. Data are expressed as mean ± SEM and were analyzed by one-way ANOVA with Tukey’s post-hoc test; n = 6.

**Table 8:** Catalytic activity of liver enzymes in animals with T2D administered compounds 3b and 4y in equimolar ratio to PGZ (15 mg/kg) for 4 weeks.

Grupo	Parameter		
	ALT/GPT (U/L)	AST/GOT (U/L)	GGT (U/L)
Control	53 ± 7	263 ± 26	10 ± 0
T2D	57 ± 12	194 ± 19	11 ± 2
T2D + PGZ	54 ± 11	285 ± 11	12 ± 1
T2D + **3b**	31 ± 2	211 ± 14	10 ± 0
T2D + **4y**	49 ± 9	236 ± 28	10 ± 0

Abbreviations: T2D: type 2 diabetes; PGZ: pioglitazone; ALT/GPT: alanine aminotransferase; AST/GOT: aspartate aminotransferase; GGT: gamma-glutamyl transferase. Data are expressed as mean ± SEM and were analyzed by one-way ANOVA with Tukey’s post-hoc test; n = 6.

**Table 9:** Distribution of animals for the evaluation of the pharmacological effect of compounds **3b** and **4y**.

Experimental Groups			
Healthy		With T2D	
Name	Treatment	Name	Treatment
Healthy without treatment (n = 6)		T2D without treatment (n = 6)	STZ, 45 mg/kg * Note 1
Healthy + Vehículo (n = 6)	Vehicle, 1 mL * Note 2	T2D + Vehicle (n = 6)	STZ, 45 mg/kg + Vehicle, 1 mL
Healthy + PGZ (n = 6)	PGZ, 15 mg/kg	T2D + PGZ (n = 6)	STZ, 45 mg/kg + PGZ, 15 mg/kg
Healthy + **3b** or **4y** (n = 6)	Compound **3b** or 4y * Note 3	T2D + **3b** or **4y** (n = 6)	STZ, 45 mg/kg + Compound **3b** or **4y**

Abbreviations: T2D: type 2 diabetes; STZ: streptozotocin; PGZ: pioglitazone; **3b**: N′-(1,3-benzothiazol-2-yl)-N-methyl(methylsulfanyl)metanimidamide; **4y**: N″-(1,3-benzothiazol-2-yl)-N,N′-di-tert-butylguanidine. * Note 1: STZ dissolved in 0.1 M citrate buffer, pH = 4.5. * Note 2: PGZ, **3b** or **4y** dissolved in water + Tween 80 + mineral oil (45:10:45). * Note 3: **3b** or **4y** administered in equimolar ratio to PGZ (15 mg/kg).

## Data Availability

All the relevant data found in the study are available in the article. The data supporting the study are in Supplementary Materials section.

## Supplementary Materials

The following supporting information can be downloaded at: https://www.mdpi.com/article/10.3390/ijms26030909/s1.
