# Supplementary material for: In Silico and In Vivo Evaluation of Novel 2-Aminobenzothiazole Derivative Compounds as Antidiabetic Agents
Source: Int J Mol Sci. 2025 Jan 22;26(3):909. doi: 10.3390/ijms26030909 (PMC11817192; doi:10.3390/ijms26030909)
Supplement: Supplementary file 1 [file ijms-26-00909-s001.zip › ijms-3344583-supplementary.pdf]

2. Results and Discussion

2.1. In Silico Studies

2.1.1. ADMET Properties

The physicochemical properties and toxicity parameters of the evaluated compounds are presented in Table S1.

Table S1. Physicochemical properties and toxicity parameters of the best benzothiazolisothioureas (**3**) and guanidinobenzothiazoles (**4**) compounds obtained from Molinspiration Cheminformatics (https://www.molinspiration.com/, accessed on 21 September 2022) [59] and DataWarrior (https://openmolecules.org/datawarrior/, accessed on 25 September 2022) [60,61].

Compound	Physicochemical properties						Toxicity (DW)				ODLS
	MW (g/mol)	cLogP	nOHNH	nON	nrotb	TPSA (Å^2)	M	T	IE	RE	
**3a**	223.33	2.41	2	3	2	51.28	X	X	X	X	1.0
**3b**	237.35	2.79	1	3	3	37.28	X	X	X	X	1.0
**3c**	251.38	3.16	1	3	4	37.28	X	X	X	√	0.96
**3d**	265.41	3.46	1	3	4	37.28	X	X	X	X	1.0
**3e**	279.43	3.97	1	3	4	37.28	X	X	X	X	1.0
**3f**	357.55	5.71	1	3	4	37.28	X	X	X	X	0.92
**3g**	299.42	4.49	1	3	4	37.28	X	X	X	X	1.0
**3h**	277.42	3.44	0	3	3	28.49	X	X	X	X	1.0
**3i**	314.44	3.92	3	4	4	63.31	X	X	X	X	1.0
**3j**	328.47	3.62	3	4	5	63.31	X	X	X	X	1.0
**3k**	314.44	3.54	3	4	4	63.31	√	X	√	X	0.92
**3l**	314.44	3.56	3	4	4	63.31	√	√	√	√	0.83
**3m**	315.42	3,98	2	4	4	57.51	X	X	X	X	1.0
**3n**	315.42	4.01	2	4	4	57.51	X	X	X	X	1.0
**3o**	343.43	4.37	5	2	5	74.58	X	X	X	X	1.0
**3p**	343.43	4.40	2	5	5	74.58	X	X	X	X	1.0
**3q**	520.73	7.01	2	6	8	74.57	X	X	X	X	0.83
**3r**	520.73	7.03	2	6	8	74.57	√	√	X	X	0.75
**3s**	498.73	5.62	0	6	6	56.99	X	X	X	X	0.92
**3t**	467.65	5.27	2	7	7	77.80	X	X	X	X	0.92
**3u**	528.80	6,82	6	2	13	74.57	X	X	X	X	0.75
**3v**	765.12	7.77	3	10	18	115.09	X	X	X	X	0.75
**3w(a)**	470.67	6.98	2	6	4	62.79	X	X	X	X	0.92
**3w(b)**	470.67	5.21	0	6	4	56.99	X	X	X	X	0.92
**4a**	192.25	2.29	4	4	1	77.30	X	X	X	X	1.0
**4b**	206.27	2.67	3	4	2	63.31	X	X	X	X	1.0
**4c**	220.30	3.04	2	4	3	49.31	X	X	X	X	1.0
**4d**	220.30	3.04	3	4	3	63.31	X	X	X	√	0.96

**4e**	234.33	3.42	2	4	4	49.31	X	X	X	√	0.96
**4f**	248.35	3.79	2	4	5	49.31	X	X	X	√	0.96
**4g**	268.35	4.36	3	4	3	63.31	X	X	X	X	1.0
**4h**	282.37	4.74	2	4	4	49.31	X	X	X	X	1.0
**4i**	296.40	5.11	2	4	5	49.31	X	X	X	√	0.87
**4j**	246.34	3.31	2	4	2	54.52	X	X	X	X	1.0
**4k**	260.37	3.69	1	4	3	40.52	X	X	X	X	1.0
**4l**	274.39	4.06	1	4	4	40.52	X	X	X	√	0.96
**4m**	300.43	4.33	0	4	3	31,73	X	X	X	X	1.0
**4n**	322.44	5.38	1	4	4	40.52	X	X	X	X	0.92
**4o**	218.28	2.21	2	4	1	49.31	X	X	X	X	1.0
**4p**	280.36	4.32	2	4	1	49.31	X	X	X	X	1.0
**4q**	281.34	3.53	1	4	1	46.52	X	X	X	X	1.0
**4r**	267.31	4.14	1	4	1	54.19	X	X	X	X	1.0
**4s**	266.33	4.04	2	4	1	56.84	X	X	X	X	1.0
**4t**	462.61	5.28	2	8	5	81.04	X	X	X	X	0.92
**4u**	290.44	4.90	2	4	5	49.31	X	X	X	X	1.0
**4v**	368.55	6.64	2	4	5	49.31	X	X	X	X	0.92
**4w**	382.58	7.15	2	4	5	49.31	X	X	X	X	0.92
**4x**	276.41	4.38	2	4	5	49.31	X	X	X	X	1.0
**4y**	304.46	5.41	2	4	5	49.31	X	X	X	X	0.92
PGZ	356.45	3.07	1	5	7	68.30	X	X	X	X	1.0

Abbreviations and definitions: **PGZ**: pioglitazone. **DW**: DataWarrior. **MW**: molecular weight ("relative" molecular mass (Mr), which is the ratio of the mass of a molecule to one-twelfth of the mass of carbon 12). **cLogP**: octanol-water partition coefficient (distribution of an organic compound between octanol and water phases, quantifying the substance's lipophilic and hydrophilic properties). **nOHNH** and **nON**: number of hydrogen bond donors and acceptors, respectively (specific type of electrostatic interaction between a proton attached to an electronegative atom (such as N or O) and a lone pair of electrons on an electronegative atom such as N, O, or F. The former is called the H-bond donor, the latter the H-bond acceptor). **nrotb**: number of rotatable bonds (movement around a metal-to-ligand bond, allowing for increased conformational flexibility in the construction of molecular assemblies with predefined shapes in coordination chemistry). **TPSA**: topological polar surface area (surface associated with heteroatoms and polar hydrogen atoms in a molecule, excluding nonpolar elements like carbon and halogens. It is a sensitive parameter influenced by the 3D conformation of the molecule and is often calculated using various methods such as dynamic averages and fragment-based increments). **M**: mutagenicity (aspect of genotoxicity manifest by mutations, i.e., heritable structural and/or numerical alterations in the DNA that irreversibly and permanently alter its information content). **T**: tumorigenicity (cancer is abnormal cell division or uncontrolled growth in response to various tumorigenic factors). **IE**: irritant effects (related to any noncorrosive substance that on immediate, prolonged, or repeated contact with normal living tissue produces a local inflammatory reaction). **RE**: reproductive effects (adverse effect on any aspect of male or female reproductive structures or functions, on the developing offspring, or on lactation, which would interfere with the development of normal offspring through sexual maturity, in turn capable of normal reproduction). √: it does present the effect; X: it does not present the effect. **ODLS**: overall drug-likeness score (sum of the molecular physicochemical properties that are characteristic of chemicals known as drugs. Indeed, drug-likeness is often used to describe pharmacokinetic and safety and can also be understood as compounds with desirable ADMET properties). The definitions of physicochemical properties and toxicity parameters were obtained from: https://www.sciencedirect.com/topics/ (accessed on 25 September 2022).

Subsequently, the compounds that resulted with a overall drug-likeness score (ODLS) of 1.0 had their toxicological properties determined using Tox-Prediction, which are presented in Table S2.

Table S2. Toxicological properties of the best benzothiazolisothioureas (**3**) and guanidinobenzothiazoles (**4**) compounds obtained from ProTox 3.0 (https://tox.charite.de/protox3/, accessed on 5 October 2022) [68,69].

Compound	LD50 (mg/kg)	Class	Hepatotoxicity	Immunotoxicity	Citotoxicity
**3a**	1190	IV	X	X	X
**3b**	1000	IV	X	X	X
**3d**	1190	IV	X	X	X
**3e**	1000	IV	X	X	X
**3g**	1000	IV	X	X	X
**3h**	1190	IV	X	X	X
**3i**	1190	IV	X	X	X
**3j**	1190	IV	X	X	X
**3m**	1190	IV	X	X	X
**3n**	1190	IV	X	X	X
**3o**	1000	IV	X	X	X
**3p**	1000	IV	X	X	X
**4a**	1190	IV	X	X	X
**4b**	1190	IV	X	X	X
**4c**	1190	IV	X	X	X
**4g**	1190	IV	X	X	X
**4h**	1190	IV	X	X	X
**4j**	1190	IV	X	X	X
**4k**	1190	IV	X	X	X
**4m**	1190	IV	X	X	X
**4o**	1190	IV	X	X	X
**4p**	1190	IV	X	X	X
**4q**	1190	IV	X	X	X
**4r**	1190	IV	X	X	X
**4s**	1190	IV	X	X	X
**4u**	1190	IV	X	X	X
**4x**	1190	IV	X	X	X
**4y**	1190	IV	X	X	X
PGZ	1000	IV	X	X	X

Abbreviations and definitions: **PGZ**: pioglitazone. **LD50**: median lethal dose (the estimated dose required to kill 50% of a group of experimental animals). **Hepatotoxicity**: Injury to the liver or impairment of the liver function caused by exposure to xenobiotics such as drugs, food additives, alcohol, chlorinated solvents, peroxidized fatty acids, fungal toxins, radioactive isotopes, environmental toxicants, and even some medicinal plants. **Immunotoxicity**: Adverse effects on the immune system caused by xenobiotics like drugs and chemicals, leading to dysfunction or structural damage, and potentially resulting in immune diseases or increased susceptibility to infections. **Citotoxicity**: Chemical potency that brings variations in cellular functions and results in cell death. X: it

does not present the toxicological effect. The definitions of toxicological properties were obtained from: https://www.sciencedirect.com/topics/ (accessed on 5 October 2022).

The ODLS obtained for each compound that oscilates in the range of 0 a 1.0, was determined from the following considerations: MW < 500 = 1, MW > 500 = 0.5; cLogP < 5 = 1, cLogP > 5 = 0.5; nON < 10 = 1, nON > 10 = 0.5; nOHNH < 5 = 1, nOHNH > 5 = 0.5; nrotb < 10 = 1, nrotb > 10 = 0.5; toxicity = 0.25 for each "it does not present the toxicological effect".

The aqueous solubility, pharmacokinetic and medicinal chemistry parameters of the compounds analyzed in SwissADME are presented in Table *S3*.

Table S3. Aqueous solubility, pharmacokinetics and medicinal chemistry parameters of the best benzothiazolisothioureas (**3**) and guanidinobenzothiazoles (**4**) compounds obtained from SwissADME (http://www.swissadme.ch/, accessed on 12 October 2022) [71,72].

Compound	Aqueous solubility			Pharmacokinetics										ODLS
	logS	Solubility (mg/mL)	Class	GI	BBB	P-gp	CYP450 inhibitor					BD	
							1A2	2C19	2C9	2D6	3A4		
**3a**	-3.00	2.24e^{-01}	III	√	X	X	√	√	X	X	X	0.55	0.84
**3b**	-3.24	1.37e^{-01}	III	√	X	X	√	√	√	X	X	0.55	0.80
**3d**	-4.08	2.23e^{-02}	II	√	X	X	√	√	√	X	X	0.55	0.56
**3e**	-4.26	1.53e^{-02}	II	√	X	X	√	√	√	X	X	0.55	0.56
**3g**	-4.93	3.52e^{-03}	II	√	X	X	√	√	√	X	√	0.55	0.52
**3h**	-4.11	2.14e^{-02}	II	√	X	X	√	√	√	X	X	0.55	0.56
**3i**	-4.57	8.51e^{-03}	II	√	X	X	√	√	√	X	√	0.55	0.52
**3j**	-4.87	4.40e^{-03}	II	√	X	X	√	√	√	√	√	0.55	0.48
**3m**	-4.78	5.21e^{-03}	II	√	X	X	√	√	√	X	√	0.55	0.52
**3n**	-4.78	5.21e^{-03}	II	√	X	X	√	√	√	X	√	0.55	0.52
**3o**	-4.77	5.86e^{-03}	II	√	X	X	√	√	√	X	√	0.55	0.62
**3p**	-4.77	5.86e^{-03}	II	√	X	X	√	√	√	X	√	0.55	0.62
**4a**	-1.95	2.15e^{+00}	IV	√	X	X	√	X	X	X	X	0.55	0.92
**4b**	-2.19	1.34e^{+00}	III	√	X	X	√	X	X	X	X	0.55	0.88
**4c**	-2.43	8.22e^{-01}	III	√	X	X	√	X	X	X	X	0.55	0.88
**4g**	-3.87	3.58e^{-02}	III	√	X	X	√	√	X	X	X	0.55	0.64
**4h**	-4.12	2.12e^{-02}	II	√	√	X	√	√	√	√	√	0.55	0.68
**4j**	-3.06	2.16e^{-01}	III	√	X	√	√	√	X	X	X	0.55	0.64
**4k**	-3.31	1.28e^{-01}	III	√	√	√	√	√	X	X	X	0.55	0.64
**4m**	-3.65	6.74e^{-02}	III	√	√	√	√	√	√	√	X	0.55	0.56
**4o**	-2.41	8.51e^{-01}	III	√	X	√	√	X	X	X	X	0.55	0.68
**4p**	-4.04	2.56e^{-02}	II	√	√	√	√	X	X	√	√	0.55	0.56
**4q**	-4.40	1.11e^{-02}	II	√	√	X	√	√	√	X	√	0.55	0.72
**4r**	-4.51	8.31e^{-03}	II	√	X	X	√	X	X	X	X	0.55	0.84
**4s**	-4.14	1.95e^{-02}	II	√	X	X	√	X	X	X	√	0.55	0.80
**4u**	-4.03	2.74e^{-02}	II	√	√	X	√	√	√	X	X	0.55	0.76
**4x**	-3.56	7.64e^{-02}	III	√	√	X	√	√	√	X	X	0.55	0.80
**4y**	-3.93	3.56e^{-02}	III	√	X	X	√	√	√	X	X	0.55	0.80

PGZ	-4.31	$1.76e^{-02}$	II	√	X	X	√	√	√	√	√	0.55	0.68

**Compound**	**Drug-likeness**					**Medicinal chemistry**		
	**Lipinski**	**Ghose**	**Veber**	**Egan**	**Muegge**	**PAINS**	**Brenk**	**Synthesis accessibility**
**3a**	√	√	√	√	√	0	2	2.83
**3b**	√	√	√	√	√	0	2	3.03
**3d**	√	√	√	√	√	0	2	3.18
**3e**	√	√	√	√	√	0	2	3.29
**3g**	√	√	√	√	√	0	2	3.26
**3h**	√	√	√	√	√	0	2	3.15
**3i**	√	√	√	√	√	0	3	3.36
**3j**	√	√	√	√	√	0	3	3.31
**3m**	√	√	√	√	√	0	2	3.21
**3n**	√	√	√	√	√	0	3	3.21
**3o**	√	√	√	√	√	0	2	3.17
**3p**	√	√	√	√	√	0	2	3.12
**4a**	√	√	√	√	X	0	2	2.50
**4b**	√	√	√	√	√	0	2	2.69
**4c**	√	√	√	√	√	0	2	2.77
**4g**	√	√	√	√	√	0	2	2.94
**4h**	√	√	√	√	√	0	2	3.05
**4j**	√	√	√	√	√	0	2	2.76
**4k**	√	√	√	√	√	0	2	2.88
**4m**	√	√	√	√	√	0	2	3.14
**4o**	√	√	√	√	√	0	1	2.74
**4p**	√	√	√	√	√	0	1	2.98
**4q**	√	√	√	√	√	0	1	3.05
**4r**	√	√	√	√	√	0	0	3.22
**4s**	√	√	√	√	√	0	0	2.86
**4u**	√	√	√	√	√	0	2	3.27
**4x**	√	√	√	√	√	0	2	3.16
**4y**	√	√	√	√	√	0	2	3.38
PGZ	√	√	√	√	√	0	1	3.46

Abbreviations and definitions: **logS**: logarithm of the aqueous solubility (key physicochemical attribute required for the characterization of an active pharmaceutical ingredient during drug discovery and beyond. Furthermore, aqueous solubility is highly important for formulation selection and subsequent development processes. **Class I**: poorly soluble; **Class II**: moderately soluble; **Class III**: soluble; **Class IV**: very soluble; **Class V**: highly soluble). **GI**: gastrointestinal absorption (related to the space that the drug can occupy in the absorption processes prior to entering the disposition space via the first pass through the liver. √: high, X: low). **BBB**: blood-brain barrier permeability (related to a physical barrier in the central nervous system that regulates the entry of molecules from the bloodstream to the brain, protecting it from harmful substances). **P-gp**: P-glycoprotein substrate (ATP-dependent cellular transporter proteins that work for the elimination of xenobiotic compounds, specifically from intracellular sites to extracellular locations across the cell membrane). **CYP450**: cytochrome P-450 (family of enzymes that play a crucial role in the metabolism of drugs and other foreign substances in the body. These enzymes carry out chemical reactions such as hydroxylation and oxidation, making them important for studying drug interactions due to the inhibition of one or more of their isoforms). **BD**: bioavailability (rate and extent to which the unchanged

drug reaches the systemic circulation and consequently at the site of action). √: it does present activity; X: it does not present activity. Lipinski's rule of five (Pfizer), Ghose filter, Veber filter, Egan filter (Pharmacia), Muegge filter (Bayer): √: it meets all parameters; X: it does not meet all parameters. **PAINS**: interference structures in panel assays (structures that may present a false positive in activity). **Brenk**: structural alert (depict fragments of compounds that could be putatively toxic, chemically reactive, and metabolically unstable). **Synthesis accessibility**: 1 easy-10 difficult. The definitions of aqueous solubility, pharmacokinetics and medicinal chemistry parameters were obtained from: https://www.sciencedirect.com/topics/ (accessed on 12 October 2022).

The ODLS obtained for each compound, which ranges from 0 to 1.0, was determined from the following considerations: logS < -5.0 = 0.5, logS > -5.0 = 1; Class V = 1, Class IV = 0.8, Class III = 0.6, Class II = 0.4, Class I = 0.2; GI√ = 1, GIX = 0.5; P-gp√ = 0, P-gpX = 1; CYP450X = 0.2 for each "it does not present activity".

2.1.2. Molecular Docking

Table S4. Binding free energy (ΔG) values of benzothiazolisothiourea ligands (**3**) and guanidinobenzothiazoles (**4**) with PPAR gamma protein obtained from PRODIGY (https://rascar.science.uu.nl/prodigy/, accessed on 7 December 2022) [81,82].

**Ligand**	**ΔG (kcal/mol)**
**3a**	-8.0
**3b**	-7.8
**4a**	-7.2
**4b**	-6.4
**4c**	-6.5
**4r**	-8.1
**4s**	-8.2
**4x**	-8.3
**4y**	-8.4
PGZ	-9.5

Abbreviations: **ΔG**: free energy of binding; PPAR gamma: peroxisome proliferator-activated receptor.

2.2. In Vivo Studies

2.2.2. Acute and Subchronic Effect of Compounds 3b and 4y in the Rat Model with T2D

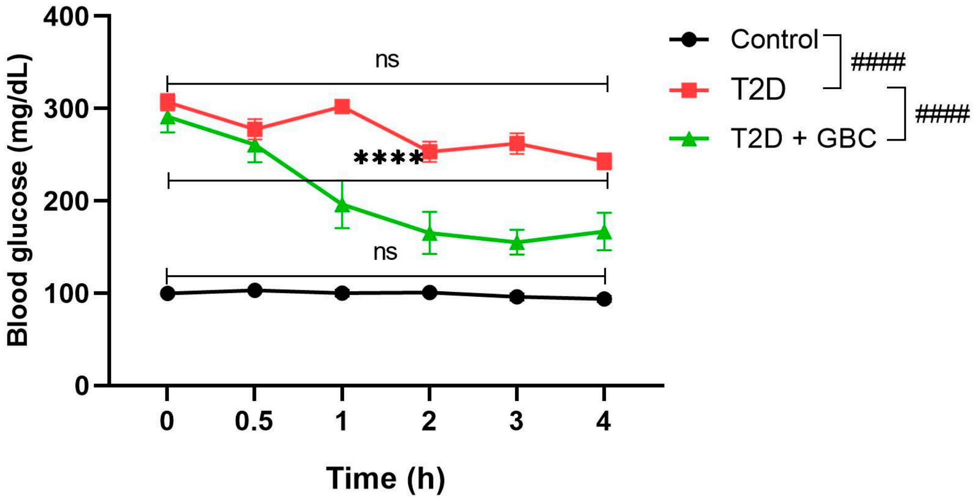

Figure S1. Blood glucose levels in animals with T2D administered a single dose of glibenclamide (GBC, 5 mg/kg). Data are expressed as mean ± SEM and were analyzed by two-way ANOVA with Tukey's post-hoc test; n = 6. The graphs represent the significant and no significant (ns) differences of each treatment group with respect to time (*0 h vs 4 h) and between them (#Control vs T2D and #T2D vs T2D + GBC) at the end of treatment (4 h). *p, #p < 0.1; **p, ##p < 0.01; ***p, ###p < 0.001; ****p, ####p < 0.0001; nsp > 0.05.

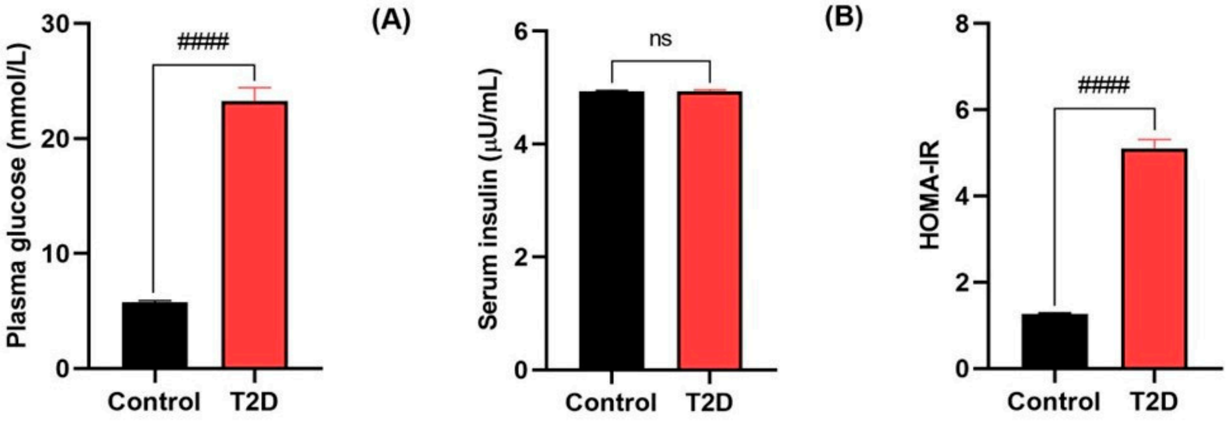

Figure S2. Plasma glucose (A), serum insulin (B), and HOMA-IR (C) levels in T2D animals administered with a single dose of STZ (45 mg/kg). Data are expressed as mean ± SEM and were analyzed by Student t-test; n = 6. The graphs represent the significant and no significant (ns) differences between treatment groups (Control vs T2D and T2D vs T2D + GBC) at the end of treatment (#4 h). *p, #p < 0.1; **p, ##p < 0.01; ***p, ###p < 0.001; ****p, ####p < 0.0001; nsp > 0.05.

3. Materials and Methods

3.3. Synthesis and Structural Identification of Compounds 3b and 4y

3.3.4. Structural Identification of Compounds 3b and 4y

methyl (E)-N'-(benzo[d]thiazol-2-yl)-N-methylcarbamimidothioate (3b)	2-(benzo[d]thiazol-2-yl)-1,3-di-tert-butylguanidine (4y)
	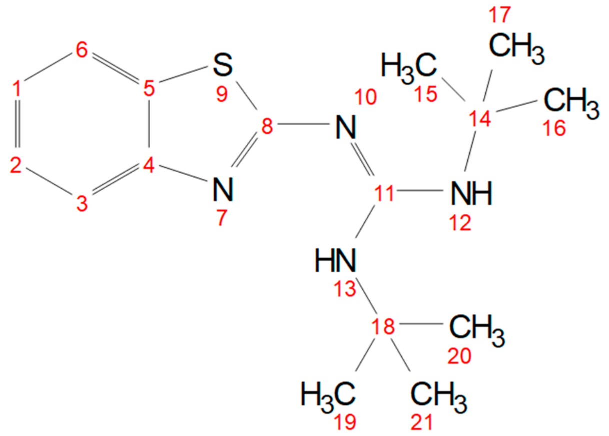

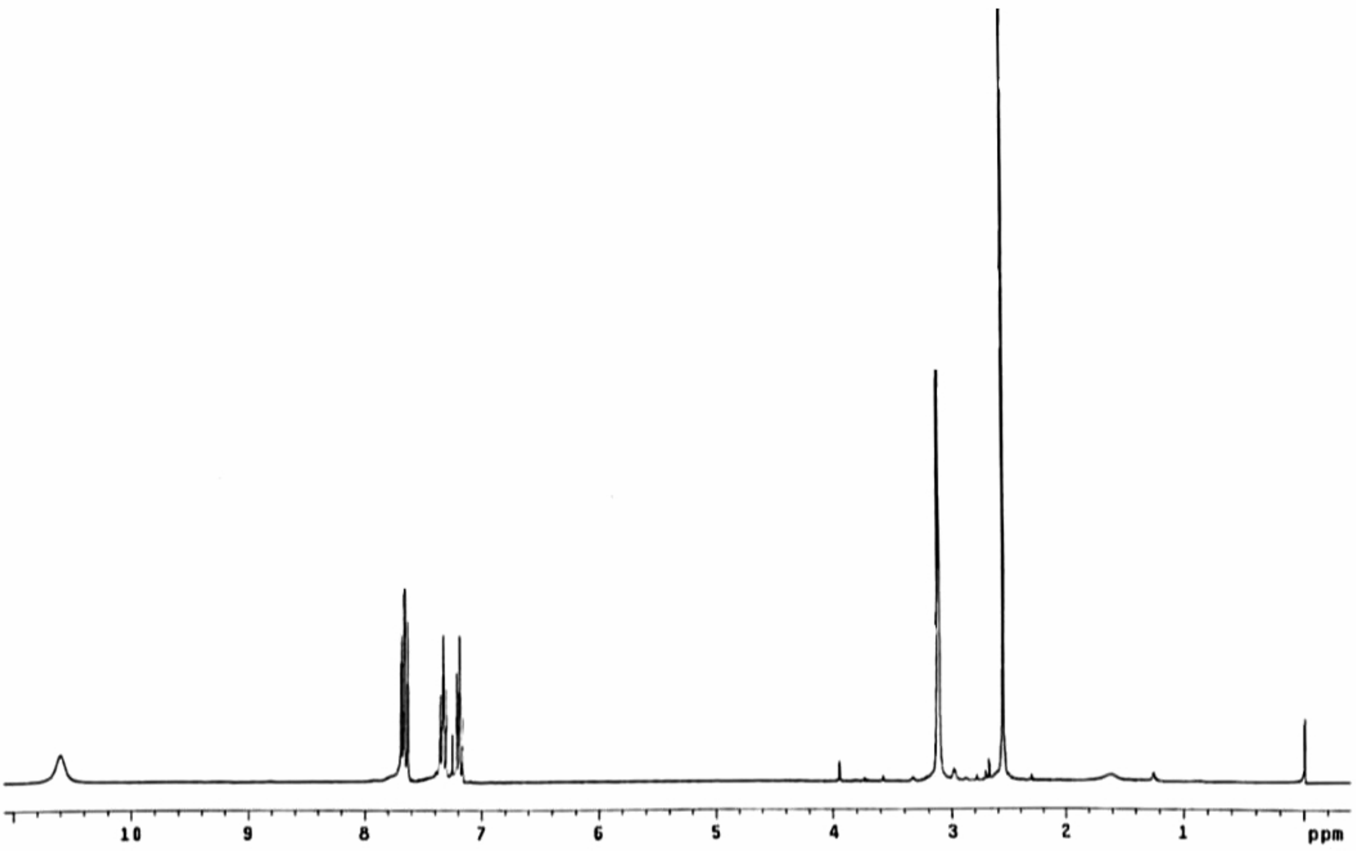

Figure S3. ^{1}H NMR spectrum of compound **3b**.

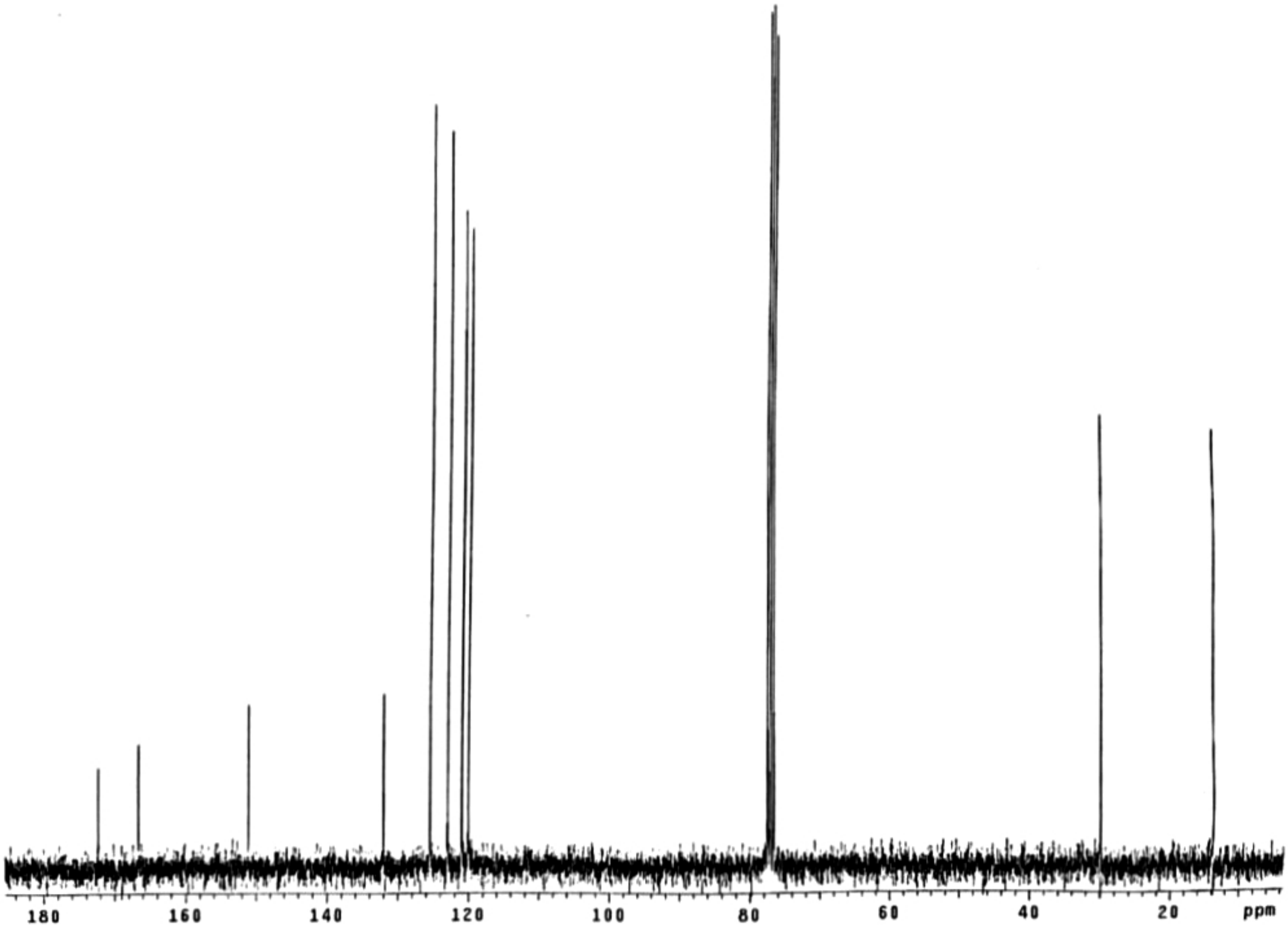

Figure S4. ^{13}C NMR spectrum of compound **3b**.

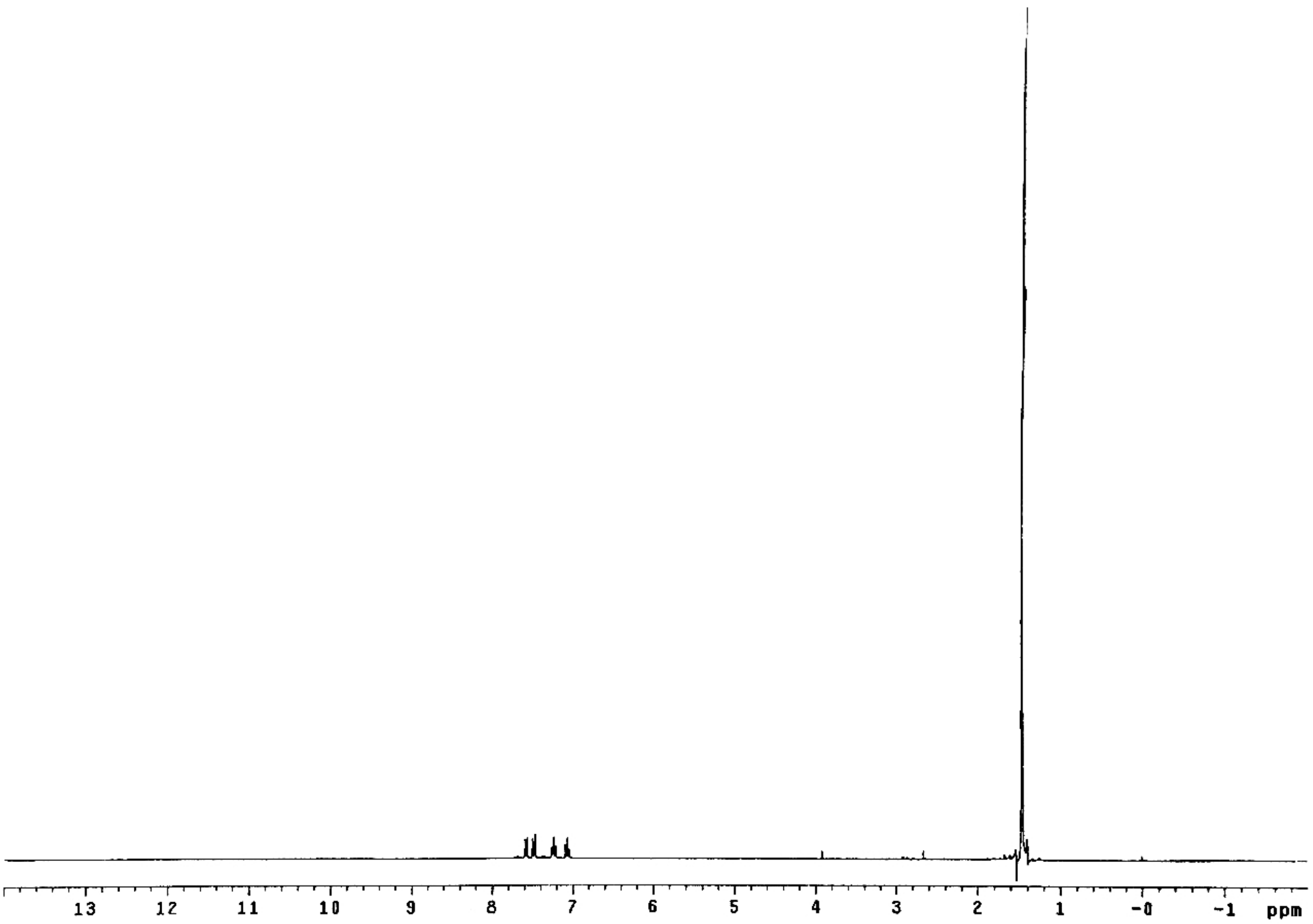

Figure S5. ^{1}H NMR spectrum of compound **4y**.

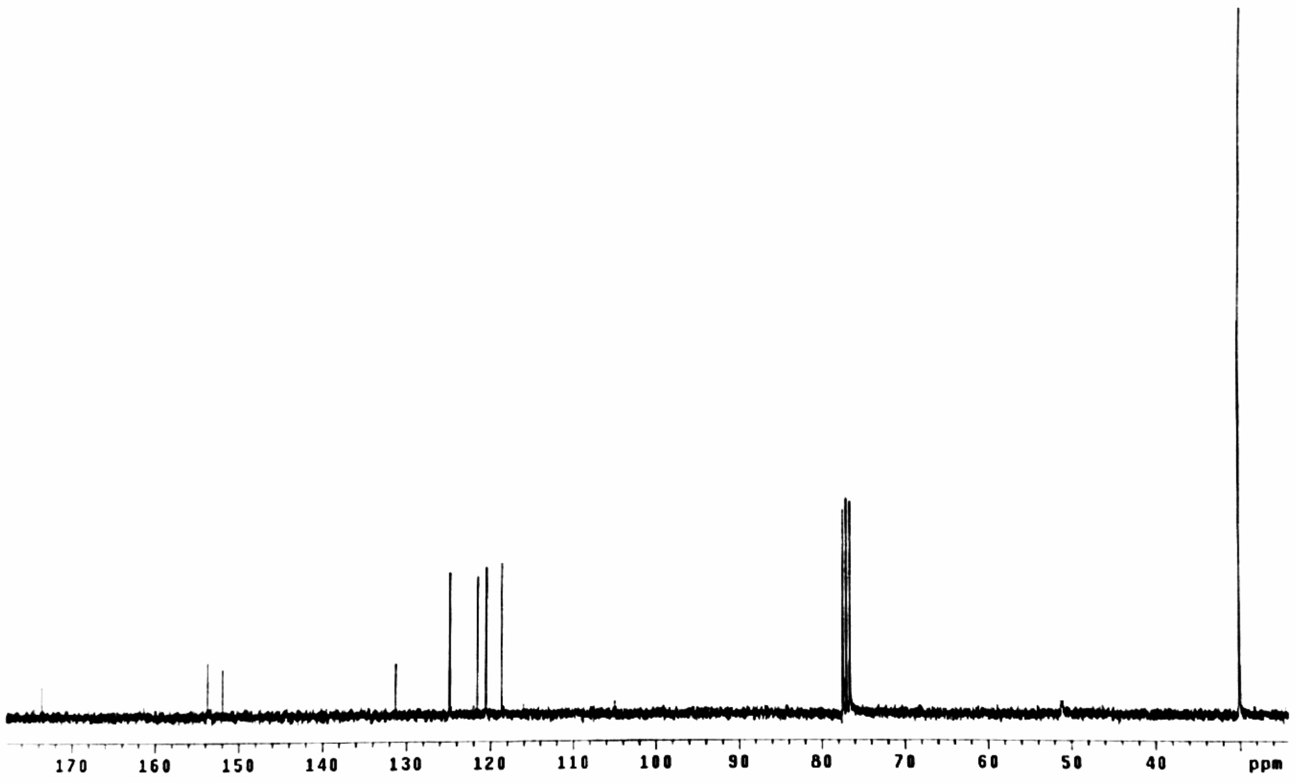

Figure S6. ^{13}C NMR spectrum of compound **4y**.

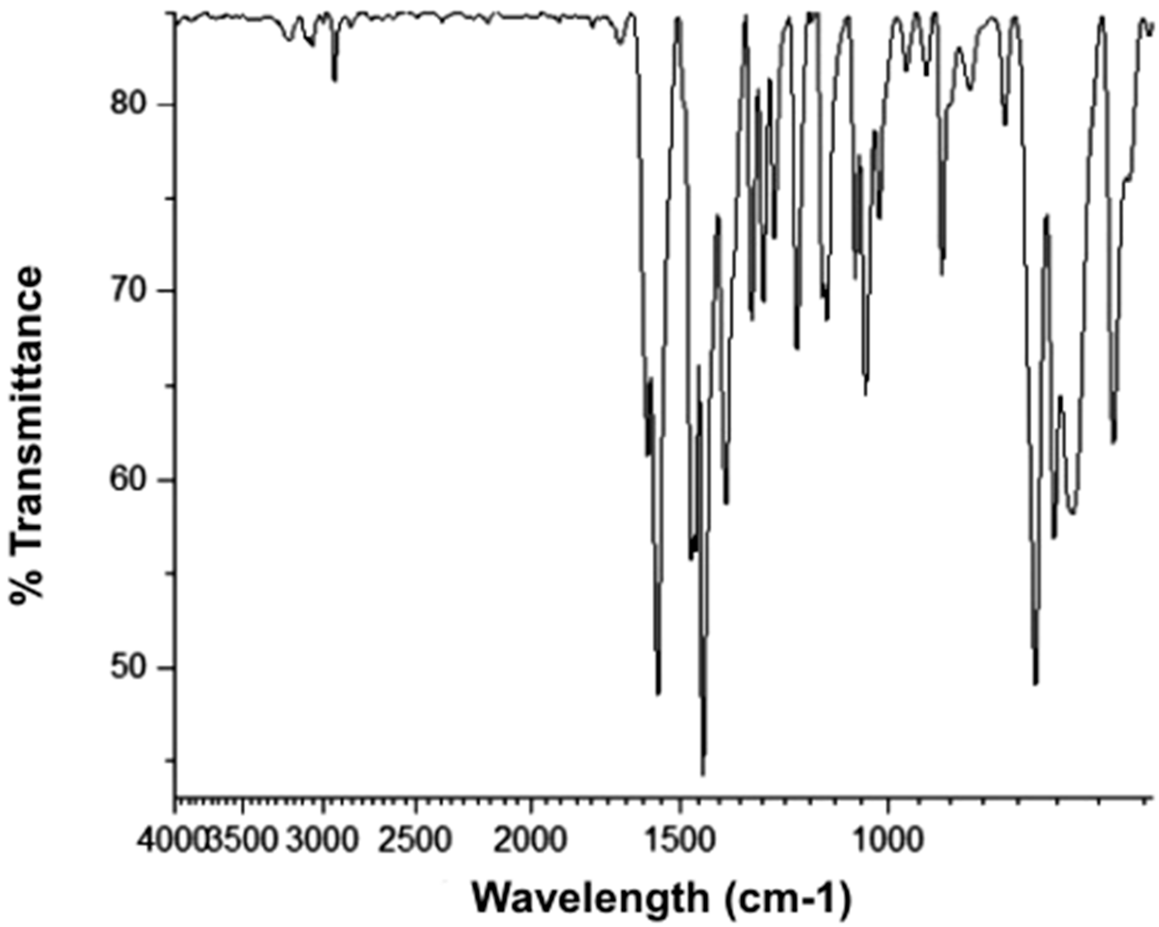

Figure S7. IR spectrum of compound **3b**.

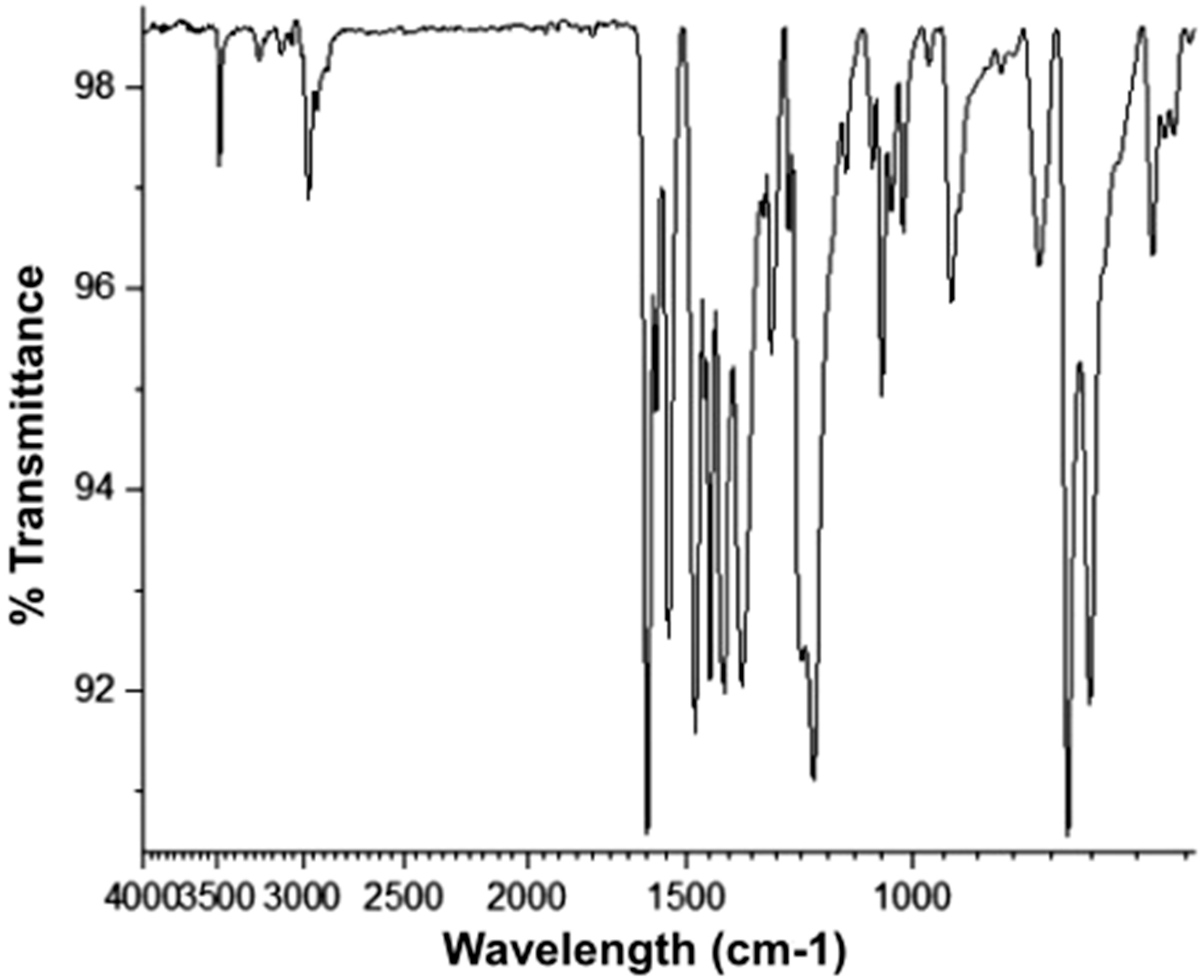

Figure S8. IR spectrum of compound **4y**.

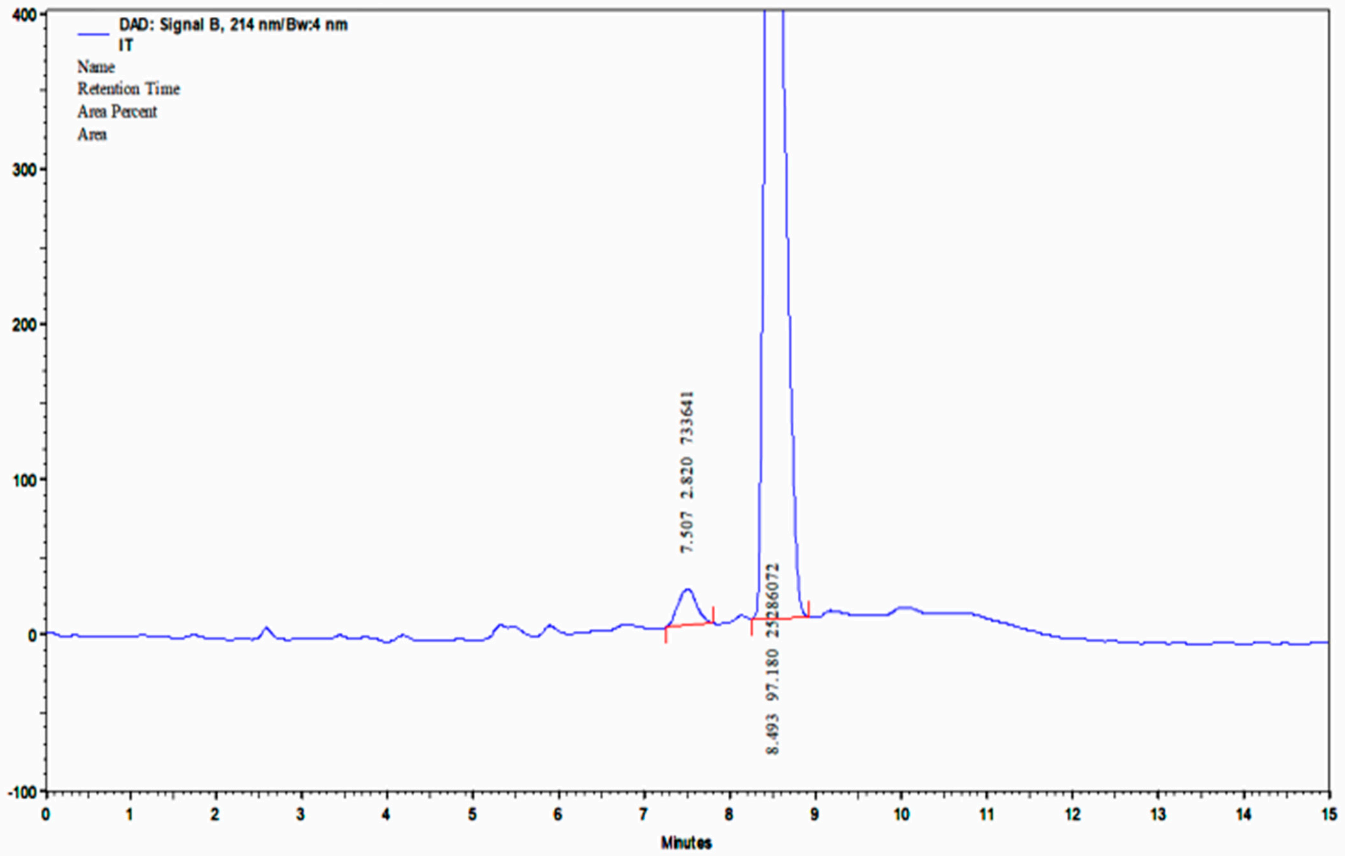

DAD: Signal B,
214 nm/Bw:4 nm
Results

Retention Time	Area	Area %	Height	Height %
7.507	733641	2.82	48491	2.28
8.493	25286072	97.18	2077092	97.72
Totals	26019713	100.00	2125583	100.00

Figure S9. HPLC analysis of compound **3b**.

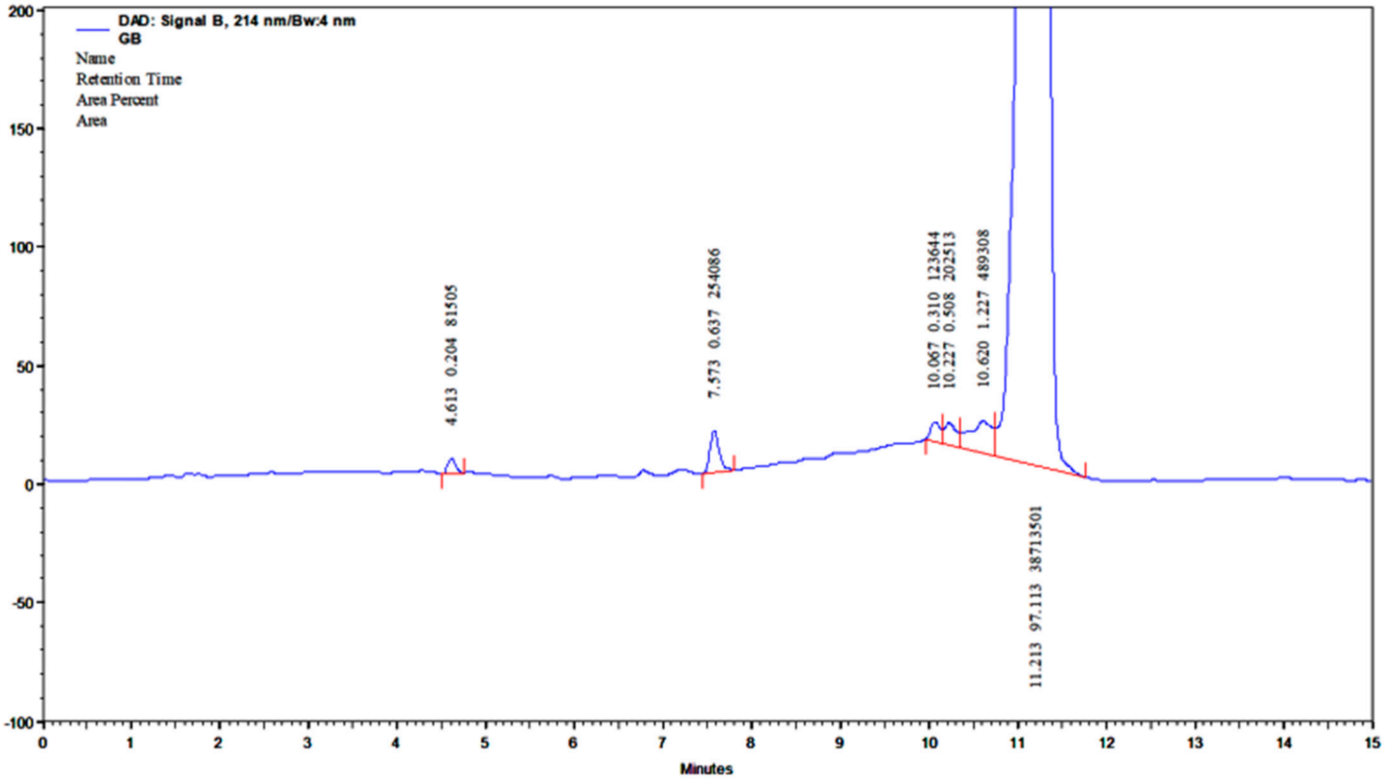

DAD: Signal B,
214 nm/Bw:4 nm
Results

Retention Time	Area	Area %	Height	Height %
4.613	81505	0.20	13687	0.57
7.573	254086	0.64	36535	1.52
10.067	123644	0.31	17486	0.73
10.227	202513	0.51	20007	0.83
10.620	489308	1.23	28512	1.19
11.213	38713501	97.11	2288925	95.17
Totals	39864557	100.00	2405152	100.00

Figure S10. HPLC analysis of compound **4y**.

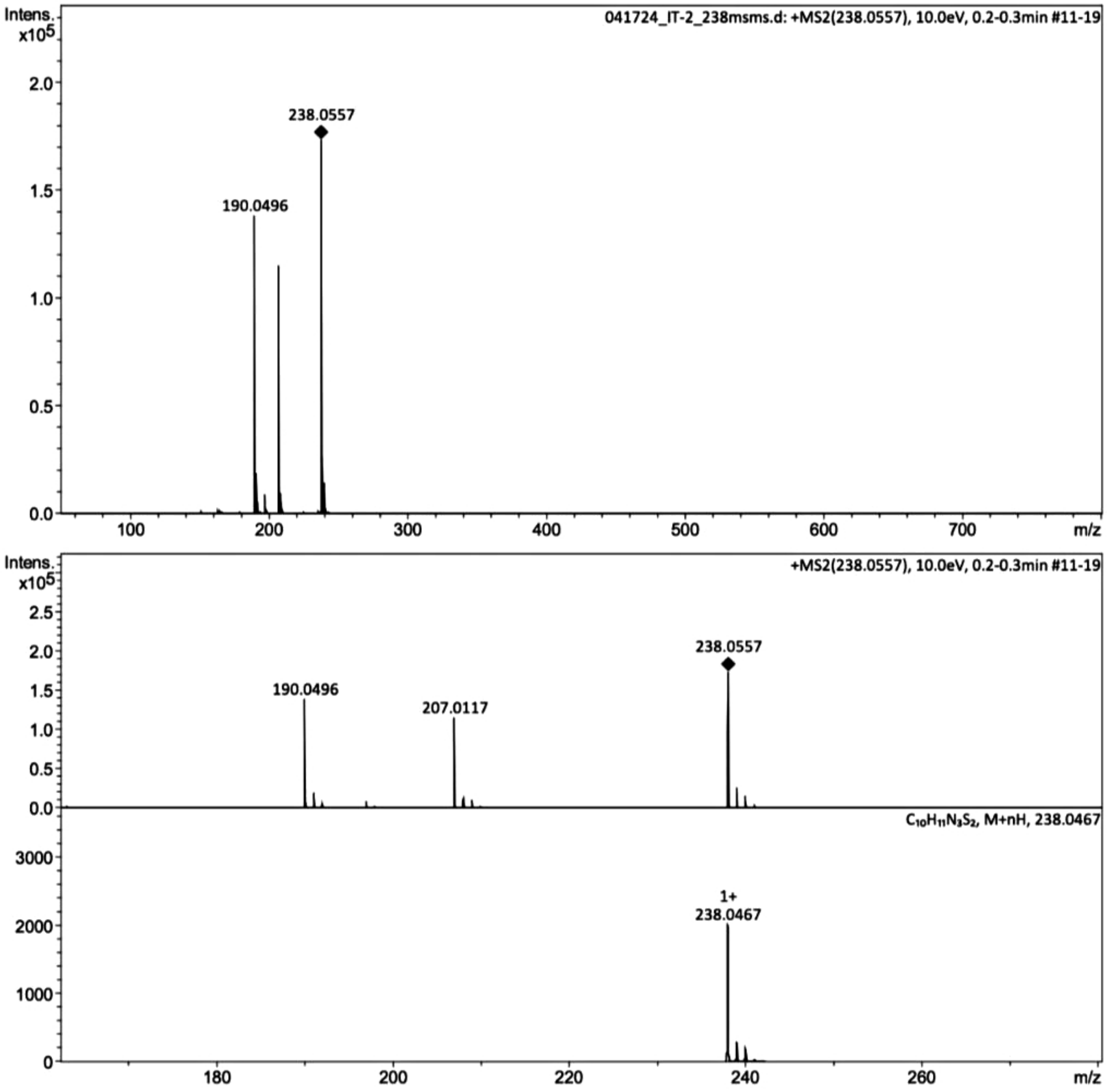

Figure S11. MS (micrOTOF-Q) spectrum of compound **3b**.

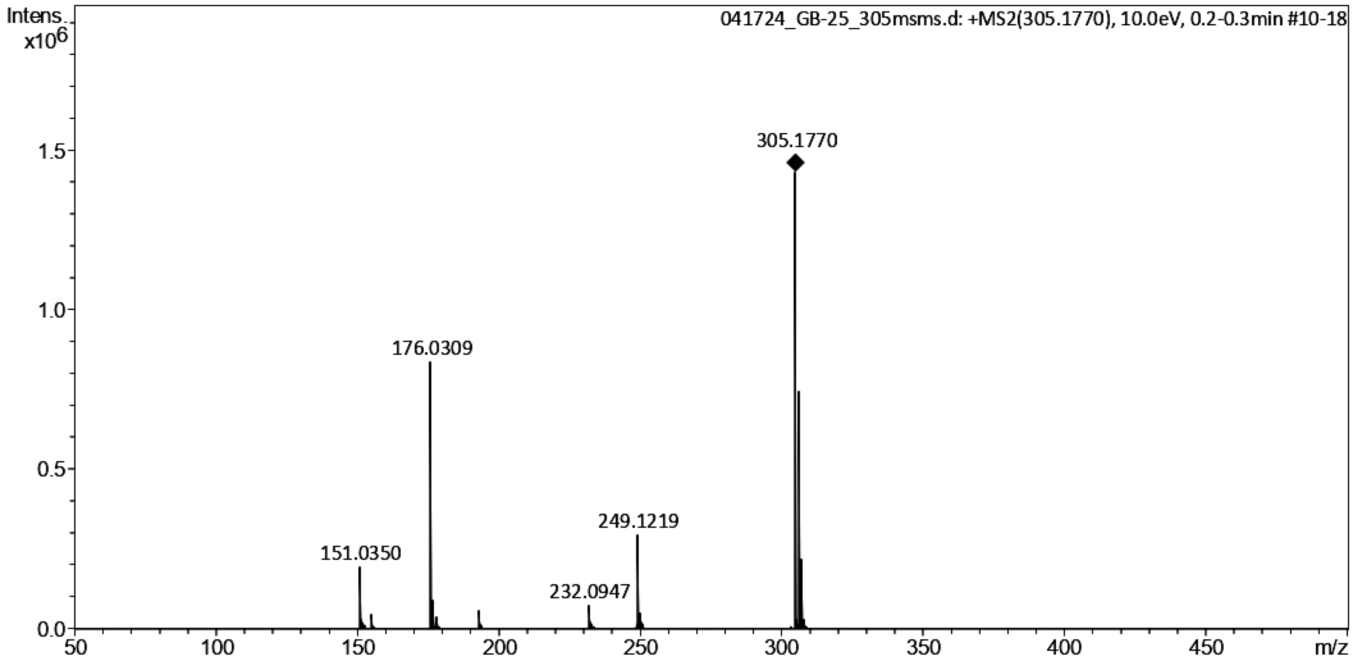

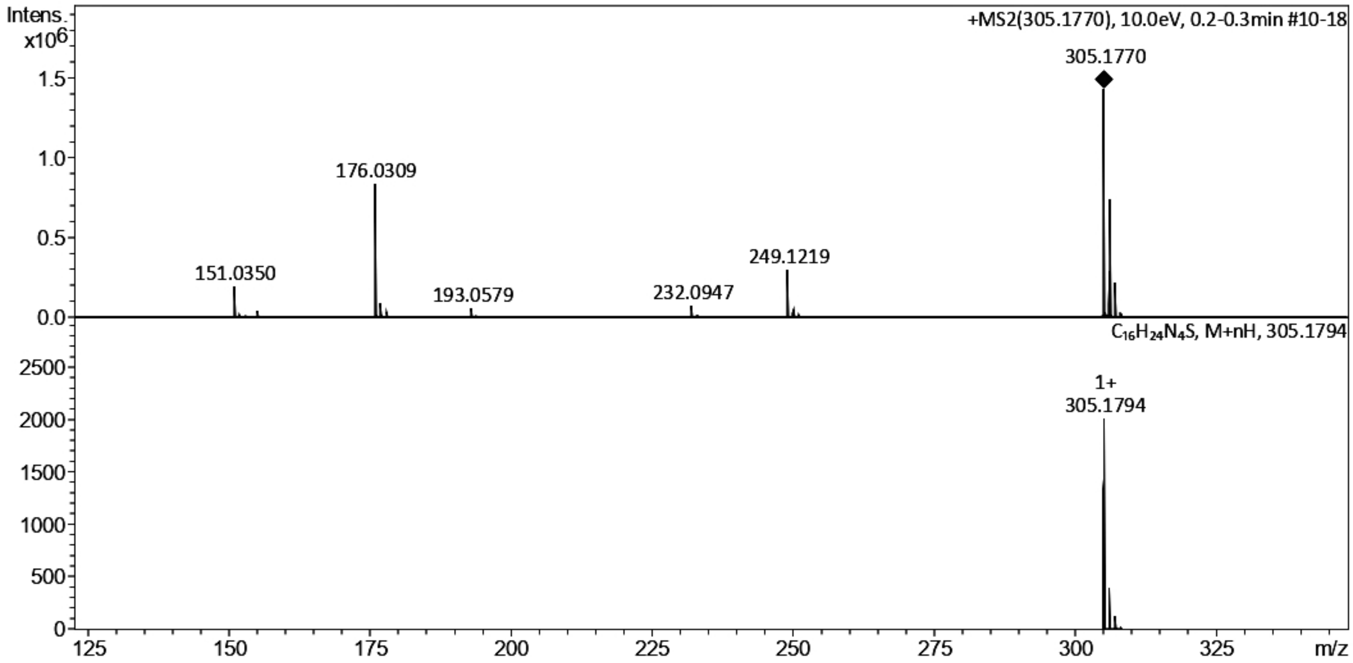

Figure S12. MS (micrOTOF-Q) spectrum of compound **4y**.
